# Association between types of antihypertensive medication and the risk of atrial fibrillation: a nationwide population study

**DOI:** 10.3389/fcvm.2024.1372505

**Published:** 2024-05-09

**Authors:** JungMin Choi, So-Ryoung Lee, Eue-Keun Choi, Kyung-Yeon Lee, Hyo-Jeong Ahn, Soonil Kwon, Bongseong Kim, Kyung-Do Han, Seil Oh, Gregory Y. H. Lip

**Affiliations:** ^1^ Division of Cardiology, Department of Internal Medicine, Seoul National University Hospital, Seoul, Republic of Korea; ^2^ Department of Internal Medicine, Seoul National University College of Medicine, Seoul, Republic of Korea; ^3^ Department of Biostatistics, College of Medicine, The Catholic University of Korea, Seoul, Republic of Korea.; ^4^ Department of Statistics and Actuarial Science, Soongsil University, Seoul, Republic of Korea; ^5^ Liverpool Center for Cardiovascular Science, University of Liverpool, Liverpool John Moores University and Liverpool Chest & Heart Hospital, Liverpool, United Kingdom; ^6^ Department of Clinical Medicine, Aalborg University, Aalborg, Denmark

**Keywords:** atrial fibrillation, hypertension, antihypertensive medication, angiotensin-converting enzyme inhibitors (ACEi), angiotensin receptor blocker (ARBs), beta blocker, calcium channel blocker (CCB), diuretics

## Abstract

**Background:**

Patients with hypertension are at a high risk of atrial fibrillation (AF). Recent research has indicated the varying effects of antihypertensive medications on developing AF.

**Objectives:**

We investigated the relationship between different types of antihypertensive medications and the risk of AF occurrence.

**Methods:**

We analyzed data from 113,582 subjects with national health screening examinations between 2009 and 2014. The study population was categorized according to antihypertensive medication type. The primary outcome was the incidence of AF.

**Results:**

Among 113,582 subjects (mean age 59.4 ± 12.0 years, 46.7% men), 93,557 received monotherapy [angiotensin receptor blockers (ARB), angiotensin-converting enzyme inhibitors (ACEi), beta-blockers, calcium channel blockers (CCB), or diuretics], while 34,590 received combination therapy (ARB/beta-blockers, ARB/CCB, ARB/diuretics, or ARB/CCB/diuretics). During a mean follow-up duration of 7.6 ± 2.1 years, 3.9% of patients were newly diagnosed with AF. In monotherapy, ACEi and CCB had similar AF risks as ARB, while beta-blockers and diuretics showed higher AF risks than ARB. In combination therapy, ARBs/CCBs and ARBs/diuretics had the lowest AF risk, whereas ARBs/beta-blockers had the highest compared to ARB/CCB. Among the specific ARBs, the AF risk varied insignificantly, except for telmisartan and candesartan.

**Conclusions:**

In hypertensive patients receiving monotherapy, ACEi and CCB showed a similar AF risk as ARBs, while beta-blockers and diuretics were associated with a higher risk. Among those receiving combination therapy, ARBs/CCBs and ARBs/diuretics had the lowest AF risk, whereas ARBs/beta-blockers showed the highest risk. Various types of ARBs have different associations with AF risk.

## Introduction

Hypertension is one of the most common comorbidities, with a prevalence of 30% among adults in 2019 (1–3). Elevated blood pressure leads to an increased risk of cardiovascular complications, such as coronary heart disease, stroke, and an increased risk of mortality (4–8). Hypertension is also a widely recognized risk factor for atrial fibrillation (AF) (9, 10). Diagnosis of hypertension, whether it is being treated or not, has been shown to increase the likelihood of AF by 70% in a previous systematic review and lowers the spontaneous restoration rate once AF is developed (11, 12). The co-prevalence of hypertension in AF patients increases the risk of major cardiovascular events and mortality compared to those without hypertension (13, 14). Based on these findings, researchers have attempted to identify the antihypertensive medication with the highest efficacy in preventing AF (15–17). The use of renin-angiotensin system inhibitors (RASi) has been associated with a 33% reduction in the risk of AF occurrence (15). The increased attention to the antiarrhythmic effects of various antihypertensive medications has led to studies comparing their differing prophylactic effects on developing AF (16, 18–21). However, the potential impact of antihypertensive medications on the risk of new-onset AF, particularly within different combinations of antihypertensive medication and specific types of angiotensin II receptor blockers (ARB), has not been addressed before.

This nationwide cohort study evaluated the effect of different antihypertensive medications using a large nationwide population.

## Methods and materials

This study utilized the comprehensive claims database from the Korean National Health Insurance Service (NHIS), which functions as the exclusive insurer for approximately 52 million individuals and corresponds to the entire population of South Korea in 2019 (22, 23). The Korean National Health Information Database (NHID) contains sociodemographic, healthcare usage, health screening, and healthcare provider data (22, 23). The National Health Screening Database provides detailed information on laboratory findings and lifestyle questionnaires (23). The healthcare utilization database comprises records of prescriptions linked with diagnoses based on the International Classification of Disease, Tenth Revision of Clinical Modification (ICD-10-CM) (22, 23).

The study was conducted following the principles of the Declaration of Helsinki. The data were anonymized; thus, the study was exempt from the Institutional Review Board (IRB) review of Seoul National University Hospital (IRB no. E-2109-118-1255). In addition, because data from the NHIS were de-identified, obtaining informed consent was not feasible. The use of the NHIS database from 2009 to 2014 will be authorized in 2023.

### Study population

**Figure 1:**
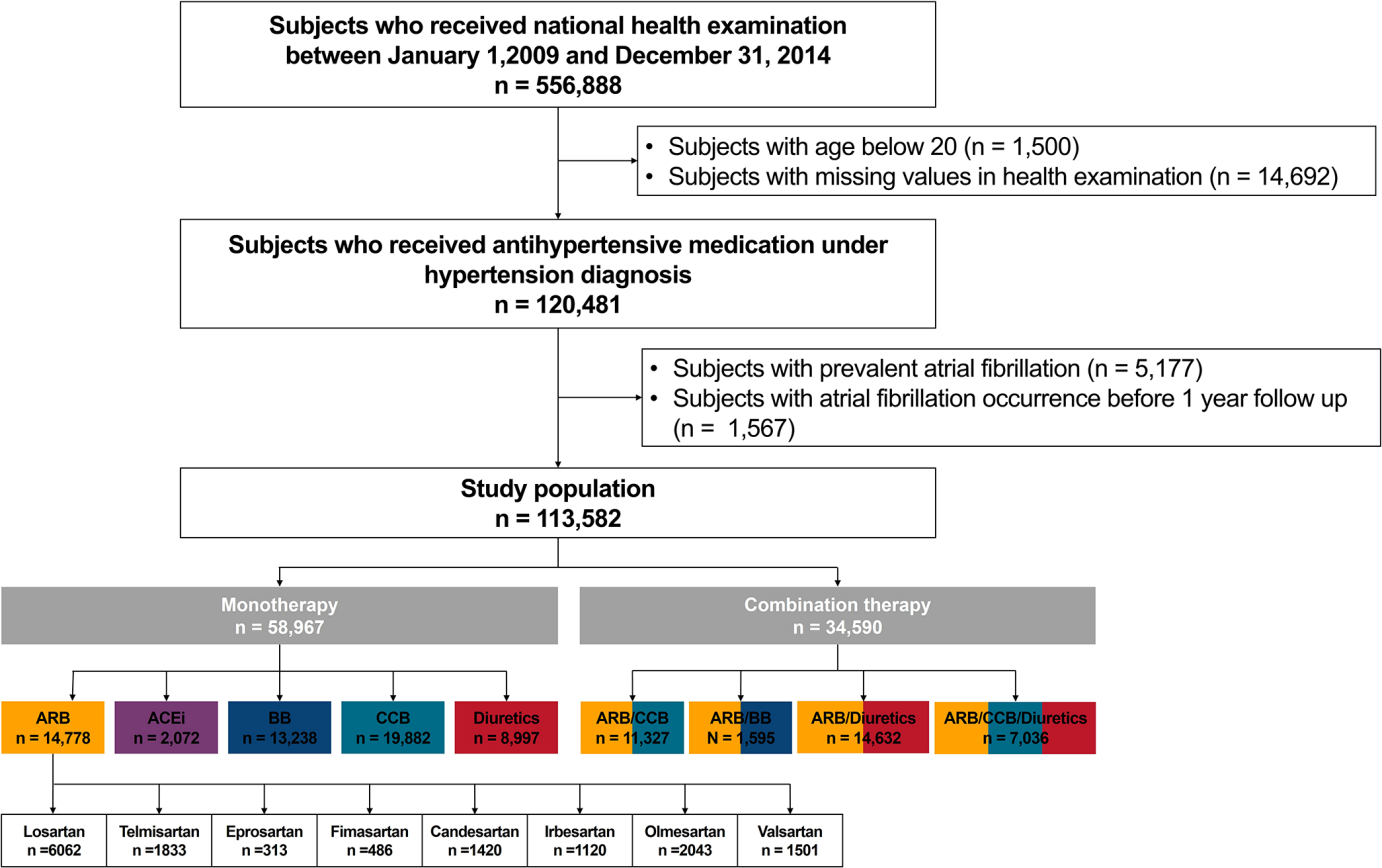
Study design. ARB, angiotensin II receptor blocker; ACEi, angiotensin-converting enzyme inhibitor; BB, beta-blocker; CCB, calcium channel blocker; NHIC, national health insurance corporation.

Figure 1 summarizes the patient flow. Subjects from the Korean National Health Insurance Service claims database who underwent screening between 1 January 2009, and 31 December 2014 (*n* = 556,888) were screened. Patients who were prescribed antihypertensive medications under hypertension diagnosis were selected (*n* = 120,481). Patients younger than 20 years (*n* = 1,500), those with preexisting AF before enrollment (*n* = 5,177), and those who developed AF before the 1-year follow-up period (*n* = 1,567) were excluded from the analysis.

The study population was by antihypertensive prescription: monotherapy [one of ARB, angiotensin-converting enzyme inhibitors (ACEi), beta-blocker, CCB, or diuretics], and combination therapy (combinations of two or three agents: ARB/CCB, ARB/beta-blockers, ARB/diuretics, and ARB/CCB/diuretics). The ARB monotherapy group was further subdivided by specific types: losartan, telmisartan, eprosartan, fimasartan, candesartan, irbesartan, olmesartan, and valsartan.

### Covariates

The covariates included patient demographics, comorbidities, medications, lifestyle, and health screening results (more details in Supplementary Table S1). The bottom 20% of NHIS participant income was deemed low. General health screening examinations collected metrics including systolic and diastolic blood pressure (SBP and DBP, respectively), weight, height, body mass index, and waist circumference measurements. Laboratory findings included fasting glucose level, estimated glomerular filtration rate (eGFR), total cholesterol, high-density lipoprotein cholesterol, low-density lipoprotein, and triglycerides (23, 24). The self-reported questionnaire included information on smoking patterns, alcohol consumption, and regular exercise (23, 25, 26).

### Study outcome and follow-up

The primary outcome was AF incidence during follow-up. AF was defined by ICD-10-CM codes (I48; AF and atrial flutter) (Supplementary Table S1) (23). The index year was the first NHIS health exam, excluding those developing AF within 1 year. Patients were monitored until AF incidence, NHIS exclusion, or study end on 31 December 2018, whichever event occurred first.

### Statistical analysis

Baseline characteristics are displayed as either mean ± standard deviation (SD) or median (interquartile range, IQR) for continuous variables and as numbers (percentages) for categorical variables, as deemed suitable. Student's *t*-tests and *χ*^2^ tests were used for continuous and categorical variables, respectively. The incidence rate (IR) was calculated as the number of events per 1,000 person-years (PY) during follow-up.

Survival analysis was performed using the Kaplan–Meier method to determine AF cumulative incidence with different antihypertensives. Cox proportional hazard regression models assessed the hazard ratio (HR) and 95% confidence intervals (CI). Four Cox models were progressively adjusted for covariates: (1) unadjusted model (model 1); (2) model adjusted for age and sex (model 2); (3) model adjusted for age, sex, comorbidities (diabetes mellitus, dyslipidemia, previous myocardial infarction, heart failure, peripheral artery disease, chronic kidney disease, stroke/transient ischemic attack, chronic obstructive pulmonary disease, thyroid disease, and sleep apnea), and social habits (smoking, alcohol consumption, regular exercise, and low income) (model 3); and (4) model 3 with addition of health screening examination measurements [SBP, fasting glucose level, total cholesterol, and body mass index (BMI)] and hypertension duration (model 4).

Subgroup analyses were conducted based on age categories (<65, 65–74, and ≥75 years), sex, obesity (BMI < 25 kg/m^2^ and ≥25 kg/m^2^), presence of abdominal obesity (men ≥ 90 cm or women ≥ 85 cm of waist circumference), smoking patterns (never, former, and current), alcohol consumption (none, mild to moderate, and heavy), income levels (low and others), and duration of hypertension (<2 years and ≥2 years). To assess the impact of antihypertensive medication on AF risk among patients without additional AF risks such as comorbidities, further sensitivity analyses were conducted on a healthy population that excluded individuals with comorbidities (diabetes mellitus, dyslipidemia, previous myocardial infarction, heart failure, prior ischemic stroke/transient ischemic attack, peripheral artery disease, chronic kidney disease, chronic obstructive pulmonary disease, and thyroid diseases).

Statistical significance was set at *p* < 0.05. All statistical analyses were performed using SAS version 9.4 (SAS Institute, Cary, North Carolina, USA).

## Results

### Study population

A total of 113,582 participants were included in the final study population. Details of the baseline characteristics for the distinct monotherapy groups are presented in Table 1, whereas those for the combination therapy group are shown in Supplementary Table S2. Specific details regarding the ARB medications are provided in Supplementary Table S3. The mean age of the total population was 59.4 ± 12.0 years old, with 46.7% men and a mean hypertension duration of 4.5 ± 3.4 years. The most prevalent comorbidity was dyslipidemia (38.4%), followed by diabetes mellitus (23.0%). Among the study population, 47.3% were obese, and 35.7% satisfied the criteria for abdominal obesity.

**Table 1:** Baseline characteristics of the study population of monotherapy.

	Total (*n* = 113,582)	Monotherapy					*p*-value
		ARB (*n* = 14,778)	ACEi (*n* = 2,072)	Beta-blockers (*n* = 13,238)	CCB (*n* = 19,882)	Diuretic (*n* = 8,997)	
Age, years							
Mean ± SD	59.4 ± 12.0	58.0 ± 11.4	60.6 ± 11.4	54.7 ± 13.7	61.6 ± 11.2	57.4 ± 13.7	<.0001
< 65	74,024 (65.2)	10,539 (71.3)	1,292 (62.4)	9,832 (74.3)	11,796 (59.3)	6,051 (67.3)	
65–74	28,470 (25.1)	3,156 (21.4)	584 (28.2)	2,534 (19.1)	5,719 (28.8)	2,014 (22.4)	
≥75	11,088 (9.8)	1,083 (7.3)	196 (9.5)	872 (6.6)	2,367 (11.9)	932 (10.4)	
Sex (men)	53,091 (46.7)	7,462 (50.5)	1,184 (57.1)	5,165 (39.0)	8,574 (43.1)	2,385 (26.5)	<0.001
Comorbidities							
Diabetes mellitus	26,107 (23.0)	4,380 (29.6)	847 (40.9)	1,607 (12.1)	3,272 (16.5)	1,160 (12.9)	<0.001
Dyslipidaemia	43,605 (38.4)	6,178 (41.8)	915 (44.2)	4,062 (30.7)	6,743 (33.9)	2,617 (29.1)	<0.001
Heart failure	4,696 (4.1)	426 (2.9)	93 (4.5)	385 (2.9)	412 (2.1)	418 (4.7)	<0.001
Prior ischemic stroke/TIA	6,220 (5.5)	880 (6.0)	163 (7.9)	500 (3.8)	1,109 (5.6)	251 (2.8)	<0.001
Prior MI	1,286 (1.1)	131 (0.9)	45 (2.2)	190 (1.4)	92 (0.5)	49 (0.5)	<0.001
PAD	19,819 (17.5)	2,281 (15.4)	369 (17.8)	1,738 (13.1)	3,507 (17.6)	1,269 (14.1)	<0.001
CKD	13,826 (12.2)	1,672 (11.3)	281 (13.6)	1,171 (8.9)	1,867 (9.4)	892 (9.9)	<0.001
COPD	10,781 (9.5)	1,256 (8.5)	194 (9.4)	1,165 (8.8)	1,950 (9.8)	1,052 (11.7)	<0.001
Sleep apnea	138 (0.1)	22 (0.2)	3 (0.1)	20 (0.2)	19 (0.1)	11 (0.1)	0.596
Thyroid disease	6,945 (6.1)	964 (6.5)	108 (5.2)	1,230 (9.3)	989 (5.0)	607 (6.8)	<0.001
HTN duration, years	4.5 ± 3.4	3.9 ± 3.3	4.8 ± 3.1	2.8 ± 3.4	4.4 ± 3.2	2.1 ± 3.1	<0.001
≥2 years	77,673 (68.4)	9,275 (62.8)	1,553 (75.0)	5,714 (43.2)	13,967 (70.3)	3,066 (34.1)	<0.001
Social history							
Smoking							<0.001
Non-smoker	75,103 (66.1)	9,406 (63.7)	1,254 (60.5)	9,252 (69.9)	13,815 (69.5)	7,003 (77.8)	
Ex-smoker	18,410 (16.2)	2,681 (18.1)	438 (21.1)	1,716 (13.0)	2,955 (14.9)	775 (8.6)	
Current smoker	20,069 (17.7)	2,691 (18.2)	380 (18.3)	2,270 (17.2)	3,112 (15.7)	1,219 (13.6)	
Alcohol consumption							<0.001
Non-drinker	71,890 (63.3)	9,122 (61.7)	1,307 (63.1)	8,902 (67.3)	13,154 (66.2)	6,496 (72.2)	
Mild to moderate							
(0–30 g/day)	33,110 (29.2)	4,528 (30.6)	630 (30.4)	3,689 (27.9)	5,368 (27.0)	2,078 (23.1)	
Heavy (≥30 g per day)	8,582 (7.6)	1,128 (7.6)	135 (6.5)	647 (4.9)	1,360 (6.8)	423 (4.7)	
Regular exercise	22,654 (20.0)	3,086 (20.9)	465 (22.4)	2,499 (18.9)	4,010 (20.2)	1,563 (17.4)	<0.001
Low income	18,259 (16.1)	2,268 (15.4)	291 (14.0)	2,120 (16.0)	3,114 (15.7)	1,620 (18.0)	<0.001
Health examination							
SBP (mmHg)	131.4 ± 16.6	132.2 ± 16.0	130.4 ± 15.7	124.6 ± 15.8	133.5 ± 15.5	123.6 ± 15.5	<0.001
DBP (mmHg)	80.6 ± 10.9	81.5 ± 10.9	79.6 ± 10.2	77.0 ± 10.4	81.7 ± 10.4	76.2 ± 10.0	<0.001
BMI (kg/m^2^)	24.9 ± 3.3	24.8 ± 3.2	24.3 ± 3.0	23.9 ± 3.2	24.5 ± 3.1	24.3 ± 3.4	<0.001
Obesity (BMI ≥ 25)	53,742 (47.3)	6,660 (45.1)	819 (39.5)	4,677 (35.3)	8,387 (42.2)	3,500 (38.9)	<0.001
WC (cm)	84.12 ± 8.86	83.83 ± 8.6	83.45 ± 8.31	80.93 ± 9.09	83.15 ± 8.27	81.31 ± 9.08	<0.001
Abdominal obesity (Men ≥ 90, Women ≥ 85)	40,575 (35.7)	4,823 (32.6)	616 (29.7)	3,308 (25.0)	6,344 (31.9)	2,624 (29.2)	<0.001
Laboratory results							
eGFR (ml/min/1.73 m^2^)	83.4 ± 35.8	83.8 ± 33.7	82.7 ± 46.6	86. 6 ± 34.6	84.4 ± 33.2	85.1 ± 27.6	<0.001
Fasting Glucose (mg/dl)	106.5 ± 31.4	109.3 ± 35.9	115.9 ± 43.3	100.5 ± 26.1	103.5 ± 27.7	100.2 ± 27.8	<0.001
Total cholesterol (mg/dl)	196.9 ± 39.4	194.8 ± 39.7	192.9 ± 39.9	195.2 ± 39.0	200.8 ± 38.5	201.3 ± 40.5	<0.001
HDL-C (mg/dl)	53.5 ± 22.7	53.5 ± 24.5	52.6 ± 18.9	54.4 ± 22.7	54.5 ± 23.1	56.3 ± 24.8	<0.001
LDL-C (mg/dl)	114.3 ± 38.6	112.9 ± 37.6	111.2 ± 36.0	114.2 ± 38.4	118.2 ± 37.1	119.1 ± 38.6	<0.001
*TG (mg/dl)	129.1 (128.7–129.5)	127.4 (126.3–128.5)	125.4 (122.5–128.3)	118.2 (117.1–119.3)	125.0 (124.1–125.9)	115.1 (113.8–116.4)	<0.001

Categorical variables are presented as percentages, and continuous variables are presented as means and standard deviations.

ACEi, angiotensin-converting enzyme inhibitor; ARB, angiotensin receptor blocker; BMI, body mass index; CCB, calcium channel blocker; CKD, chronic kidney disease; COPD, chronic obstructive pulmonary disease; DBP, diastolic blood pressure; eGFR, estimated glomerular filtration rate; FLI, fatty liver index; HDL-C, high-density lipoprotein cholesterol; LDL-C, low-density lipoprotein cholesterol; MI, myocardial infarction; PAD, peripheral artery disease; SBP, systolic blood pressure.

*TG is presented as the geometric mean (95% confidence interval).

### Risk of incident AF according to specific antihypertensive medication

**Figure 2:**
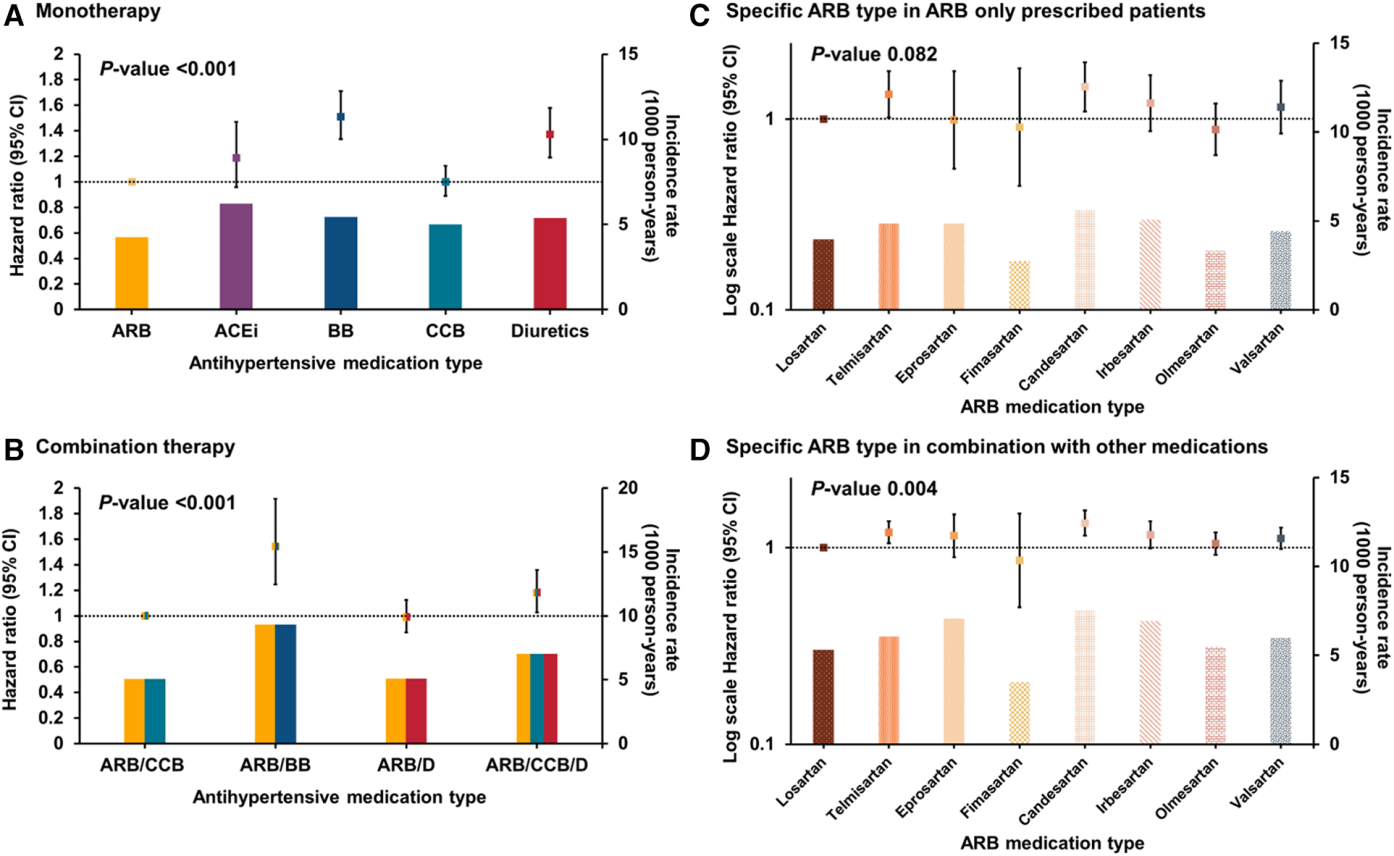
Hazard ratio and incidence rate comparison in each antihypertensive medication group; (**A**) monotherapy group (**B**) combination therapy group (**C**) specific ARB type in ARB only prescribed group (**D**) specific ARB type in combination with other medication group. Model 4: model adjusted for age, sex, comorbidities (diabetes mellitus, dyslipidemia, previous myocardial infarction, heart failure, peripheral artery disease, chronic kidney disease, stroke/transient ischemic attack, chronic obstructive pulmonary disease, thyroid disease, and sleep apnea), and social habits (smoking, alcohol consumption, regular exercise, and low income), health screening examination measurements (SBP, fasting glucose level, total cholesterol, and BMI) and hypertension duration. ARB, angiotensin II receptor blocker; ACEi, angiotensin-converting enzyme inhibitor; BB, beta-blockers; CCB, calcium channel blocker; CI, confidence interval; HTN, hypertension.

During a mean follow-up duration of 7.6 ± 2.1 years, AF was newly diagnosed in 3,741 (3.9%) patients. The adjusted HR (aHR) with 95% CI and IR according to the type of antihypertensive agent used in patients receiving antihypertensive monotherapy is shown in Figure 2A and Supplementary Table S4. In those on monotherapy, subjects administered beta-blocker exhibited the highest increase in AF risk (aHR, 1.51; 95% CI, 1.33–1.71, *p *< 0.001), followed by diuretics (aHR, 1.37; 95% CI, 1.19–01.58, *p *< 0.001), when compared to those receiving ARBs. Subjects taking ACEi or CCBs showed comparable risk of AF with ARBs (aHR, 1.19; 95% CI, 0.96–1.47 for ACEi, and aHR, 1.00; 95% CI, 0.89–1.12 for CCB, respectively, *p *< 0.001).

Figure 2B shows the aHR (95% CI) and IR based on a particular type of antihypertensive medication in the combination therapy group. Comprehensive details are provided in Supplementary Table S4.

Within the population receiving combination antihypertensive therapy, individuals prescribed ARB/beta-blockers exhibited the highest risk (aHR, 1.54; 95% CI, 1.25–1.92, *p *< 0.001), followed by ARB/CCB/Diuretics with an aHR of 1.18, 95% CI, 1.03–1.36 (*p* < 0.001) compared to those taking ARB/CCB. Patients with ARB/diuretics did not show a significant difference in AF risk compared to those taking ARB/CCB (adjusted HR, 0.99; 95% CI, 0.87–1.12, *p *< 0.001).

Figure 2C illustrates the aHR (95% CI) and IR for specific ARB types in patients who were exclusively prescribed ARB monotherapy. Figure 2D presents the same data for patients receiving ARBs in combination therapy. Additional information is provided in Supplementary Table S5.

In the subset of individuals receiving ARB monotherapy, most ARB medications exhibited no notable variation in AF risk compared with losartan. In the population receiving ARB as part of antihypertensive combination therapy, when compared to losartan, candesartan and telmisartan were associated with a higher risk of AF (aHR, 1.33; 95% CI, 1.15–1.54, and 1.20; 95% CI, 1.05–1.36) (*p *= 0.004). Other ARBs did not show significant differences compared to losartan.

**Figure 3:**
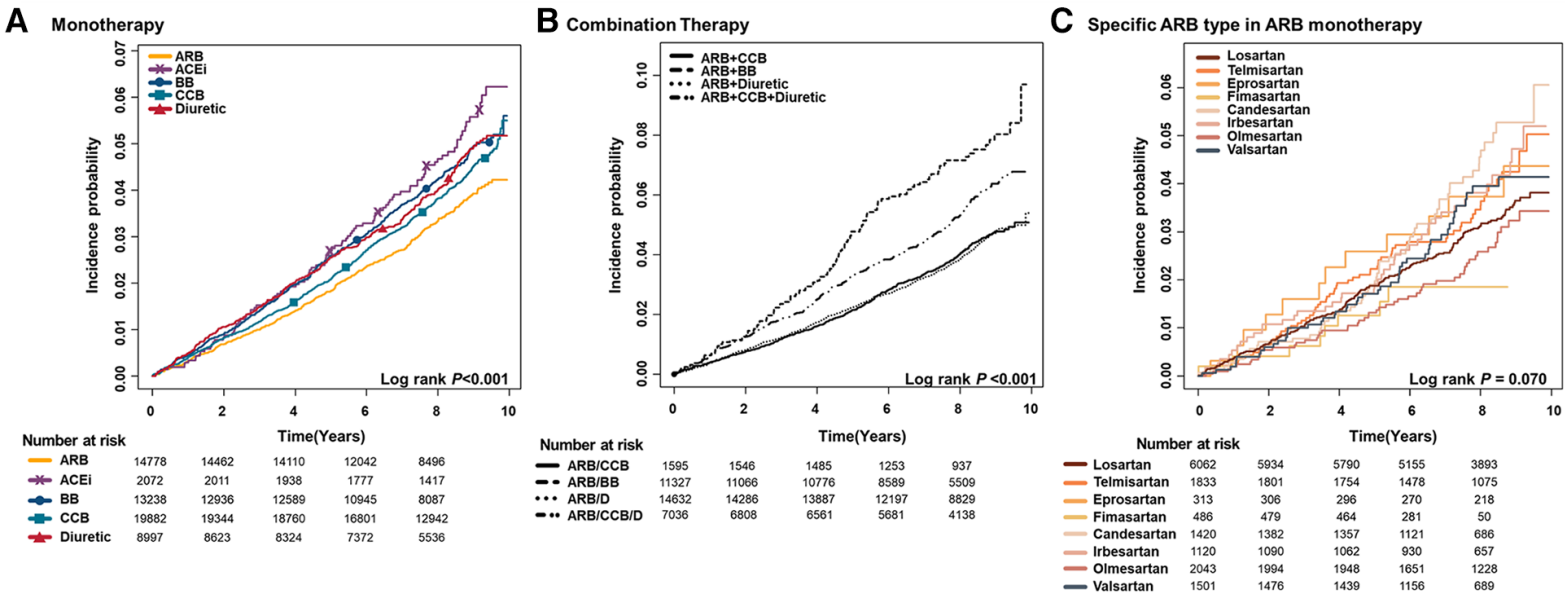
Cumulative incidence curves of AF stratified by antihypertensive medication; (**A**) monotherapy (**B**) combination (**C**) specific type of ARB in ARB monotherapy patients. ARB, angiotensin II receptor blocker; ACEi, angiotensin-converting enzyme inhibitor; BB, beta-blocker; CCB, calcium channel blocker; D, diuretic.

Figure 3 illustrates the cumulative incidence of AF with distinct antihypertensive medications.

### Subgroup analyses

Subgroup analyses conducted for ARB monotherapy, combination therapy, and specific ARB types are provided in Supplementary Tables S6–S8, respectively.

In the monotherapy group, the different effects of antihypertensive medication types on AF risk were notably attenuated among individuals aged >75 years (*p for interaction *= 0.034). No significant interactions were observed in the other subgroups, including sex, body mass index, abdominal obesity, smoking habits, alcohol consumption, income level, SBP, and duration of hypertension. Within the combination therapy group, in obese patients, compared to ARB/CCB, the relative AF risk of ARB/CCB/diuretics was accentuated (aHR, 1.30; 95% CI, 1.09–1.56, *p *= 0.007). A similar pattern was observed for those with abdominal obesity (aHR, 1.34; 95% CI, 1.10–1.63, *p *= 0.014) and SBP over 130 mmHg (aHR, 1.34; 95% CI, 1.13–1.60, *p *= 0.012). Subgroup analyses of specific ARB types within the ARB monotherapy group did not indicate significant interactions across subgroups.

### Sensitivity analyses

In the sensitivity analyses of monotherapy group, subjects taking ACEi, beta-blocker, and diuretics were associated with a higher risk of AF compared to ARB (aHR, 2.40; 95% CI, 1.41–4.08 for ACEi, aHR, 1.85; 95% CI, 1.34–2.55 for beta-blocker, and aHR, 1.59; 95% CI, 1.12–2.27 for diuretics, *p *< 0.001) (Supplementary Table S8). In the combination therapy group, the result was consistent with the main results, showing ARB/beta-blocker to exhibit the highest AF risk compared to ARB/CCB (aHR, 2.75; 95% CI, 1.44–5.24, *p *= 0.023). Sensitivity analysis of specific ARB types is shown in Supplementary Table S9. ARB, in combination with other antihypertensive medications, did not show a significant difference in the risk of AF occurrence (*p *= 0.596).

## Discussion

The main findings of this study are as follows: (1) In the hypertensive patient group receiving antihypertensive monotherapy, those treated with ACE inhibitors and CCBs showed a similar AF risk to those treated with ARBs, while those treated with beta-blockers and diuretics showed a higher AF risk; (2) In the group receiving combination therapy, those treated with ARB/diuretics and ARB/CCB/diuretics showed a similar AF risk to those treated with ARB/CCBs, while those treated with ARB/beta-blockers showed a higher AF risk; and (3) Among the specific types of ARBs, the AF risk did not differ significantly except for telmisartan and candesartan in both the monotherapy group and the combination therapy group.

The protective effect of RASi on the risk of AF occurrence for both primary and secondary prevention has been shown in previous studies with other ethnicities (15, 17–19, 27). The Losartan Intervention For End Point Reduction in Hypertension (LIFE) trial showed that losartan intervention was associated with a lower incidence of new-onset AF and associated stroke than atenolol (20).

Comparisons between RASi and diuretics or beta-blockers have been conflicting in previous studies, with randomized clinical trial data only available for ACEis and not ARBs (16, 28, 29). Previous randomized clinical trials have failed to show significant benefits of ACEi compared with diuretics and beta-blockers in AF risk (28, 29). However, a recent nationwide population study on both ACEi and ARB showed a lower incidence of AF in patients prescribed RASi compared with beta-blockers or diuretics (16). This difference may be attributed to the specific type of RASi used in the clinical trial, as we observed slight variations in AF risk among different subtypes of ARBs in our study.

The antiarrhythmic effect on developing AF was most prominent among individuals with left ventricular hypertrophy or heart failure (30). The beneficial effect of RASi on developing AF is thought to be attributable to atrial electrical remodeling of RASi, as seen in animal models (31, 32). Furthermore, recent studies have compared the effect of angiotensin receptor-neprilysin inhibitor (ARNI) with ARB and found a better reduction of atrial electrical instability in the ARNI group in a retrospective study and animal experiment (33). However, there is a limitation in the generalizability of the result, as ARNI is only recommended for heart failure management and not hypertension in current guidelines, and further research is needed (34).

As a result of previous studies on antihypertensive medication, RASi has been stated in the European Society of Cardiology AF management guidelines as an upstream therapy among patients with left ventricular dysfunction, left ventricular hypertrophy, or hypertension (35). Also, as stated in management guidelines, based on a holistic or integrated care management approach, hypertension control is very important for patients who are already diagnosed with AF (35, 36). The coexistence of hypertension in patients with AF increases the risk of stroke, and guidelines emphasize attention to good BP control in AF patients with hypertension to reduce AF and the risk of stroke and bleeding (35). Indeed, adherence to the Atrial fibrillation Better Care (ABC) pathway is associated with improved clinical outcomes (37, 38).

In this study, the use of beta-blockers was associated with a higher risk of AF than ARB monotherapy or ARB/CCB combination therapy. Also, a recent study found the beta-blocker usage to be associated with impaired left atrial function in patients with hypertension and without heart failure or AF (39). In the course of hypertension, uncontrolled blood pressure was associated with reduced early diastolic filling, atrial remodeling, and increased AF inducibility (40, 41). The difference in the preventative effects of atrial remodeling between RASi and beta-blockers may have led to unfavorable outcomes in terms of AF and stroke risk among beta-blocker users compared to RASi users as seen in previous studies (16, 20). Additionally, the prescription of beta blockers to patients with hypertension may be due to suspicion of undiagnosed AF.

In line with the findings of a previous Danish study, our study also observed a higher risk of AF among individuals prescribed diuretics (16). This increased risk may be attributable to the fact that the prescription of diuretics in patients with hypertension could be due to a symptomatic prescription of heart failure that has not yet been diagnosed. While our study found no benefits of diuretics in general hypertensive patients, meta-analyses of heart failure patients suggest that mineralocorticoid receptor blockade may have the potential to reduce AF risk (42). A recent study on a novel, selective, nonsteroidal mineralocorticoid receptor antagonist, finerenone, also found potential for reducing AF risk in patients with chronic kidney disease and type 2 diabetes (43).

To our knowledge, this study is the first to compare the AF risk among different types of ARBs. Previous studies have focused on the comparison between ARBs and placebo or other types of antihypertensive medications, such as diuretics or beta-blockers, for new-onset AF (18–20). The head-to-head comparison of the different types of ARBs has not yet been performed. Although this study had a retrospective design, it is novel in exploring the differences between ARBs in incident AF risk. In this study, most ARBs showed similar AF risks to losartan, except for telmisartan and candesartan, which showed a higher AF risk. These drugs have both been shown to decrease AF risk compared to *placebo* in previous studies (18, 19). However, caution is needed when interpreting the results, as our study used losartan as a reference and not a placebo. The variation among different types of ARBs may be due to their differing binding affinities, lipophilicities, and metabolism (44). For example, candesartan and telmisartan are known to have the highest binding affinity to AT1 receptor antagonist (45). Telmisartan has distinct characteristics such as partial agonistic effect on the peroxisome proliferator-activated receptor and stronger inhibitory effects on arachidonic acid (46, 47). Candesartan is the only drug among ARBs to be a prodrug and is converted during gastrointestinal absorption (48). These unique characteristics of candesartan and telmisartan might have led to a different effect on AF risk. Furthermore, differences in the study population should also be considered. For example, the TRANSCEND trial (the Telmisartan Randomized AssessmeNt Study in ACE iN-tolerant subjects with cardiovascular disease) and CHARM (the Candesartan in Heart Failure: Assessment of Reduction in Mortality and Morbidity) focused on high-risk patients with cardiovascular disease or diabetes with end-organ damage or symptomatic congestive heart failure, respectively (18, 19).

However, further prospective research will be needed to reach a definitive conclusion.

### Study limitations

This study has several limitations. First, AF may have been underestimated in this study because the definition of AF was limited to the ICD-10-CM code diagnosis without a review of electrocardiograms. However, using ICD-10-CM codes for AF diagnosis has shown a predictive value of 94.1% in a previous study (49). Second, only blood pressure at baseline was included, and information on blood pressure control was not considered. The degree of high blood pressure showed a linear increase in systolic blood pressure and AF risk (50). Thus, controlling hypertension with antihypertensive medications may have affected the incidence of AF. Third, the specific dose of antihypertensive medication or the echocardiographic data of the patients could not be evaluated. This limitation was due to the nature of the NHID used in this study. The current NHID dataset does not provide the specific doses of antihypertensive medications or echocardiographic data. Further research is required to compare the effects of different doses of antihypertensive medications. Fourth, changes in antihypertensive medication or diagnosis of new comorbidities could not be accounted for, as only the baseline values were retrieved. These changes may have affected the incidence of AF. Fifth, beta-blockers and diuretics are not only used for hypertension alone but also for other purposes, such as heart failure. Thus, the influence of comorbidities not reported in the NHID (such as impending heart failure) on the incidence cannot be ignored. However, because the sensitivity analyses showed a consistent trend, such confounders' effect was presumed negligible.

## Conclusion

In monotherapy, ACEi and CCB exhibited a similar AF risk to that of ARB, while beta-blockers and diuretics were associated with a higher risk. Among those receiving combination therapy, the lowest AF risk was observed with ARBs/CCBs and ARBs/diuretics, whereas ARBs/beta-blockers had the highest risk. Various types of ARBs exhibit varying degrees of association with the risk of AF, suggesting that there may be a nuanced relationship between different types of ARBs and the likelihood of developing AF.

## Data Availability

The datasets presented in this article are not readily available due to legislation by the Korean government. Requests to access the datasets should be directed to Korean National Health Insurance Service.
