# Supplementary material for: Association between types of antihypertensive medication and the risk of atrial fibrillation: a nationwide population study
Source: Front Cardiovasc Med. 2024 May 9;11:1372505. doi: 10.3389/fcvm.2024.1372505 (PMC11111936; doi:10.3389/fcvm.2024.1372505)
Supplement: Supplementary file 1 [file Table1.docx]

Supplemental Material

**Supplemental Tables**

**Table S1.** Definitions of covariates

**Table S2.** Baseline characteristics of the study population of combination therapy

**Table S3**. Baseline characteristics of the study population according to specific ARB Type

**Table S4**. Hazard ratios for atrial fibrillation according to the antihypertensive medication type

**Table S5**. Hazard ratios for atrial fibrillation according to the specific ARB type

**Table S6**. Subgroup analyses according to antihypertensive medication types

**Table S7**. Subgroup analyses according to specific ARB type in ARB monotherapy

**Table S8.** Sensitivity analyses of antihypertension medications: analyses on subjects without significant comorbidities

**Table S9.** Sensitivity analyses of specific ARB type

**Supplemental Tables**

**Table S1. Definitions of covariates**

| **Diagnosis** | **ICD-10-CM code and definition** | **Diagnostic definition** |
| --- | --- | --- |
| **Inclusion/exclusion criteria** |  |  |
| **Atrial fibrillation** | I48.0-48.4, I48.9 | Admission ≥ 1 or outpatient department ≥ 2 |
| **Hypertension** | I10-I13, I15; and minimum 1 prescription of anti-hypertensive drug (thiazide, loop diuretics, aldosterone antagonist, alpha-/beta-blocker, calcium-channel blocker, angiotensin-converting enzyme inhibitor, angiotensin II receptor blocker). | Admission≥1 or outpatient department≥1 |
|  | Or systolic/diastolic blood pressure ≥ 140/90 mmHg | Index health examination |
| **Comorbidities** |  |  |
| **Diabetes mellitus** | E11-E14; and minimum 1 prescription of anti-diabetic drugs (sulfonylureas, metformin, meglitinides, thiazolidinediones, dipeptidyl peptidase-4 inhibitors, α-glucosidase inhibitors, and insulin). | Admission≥1 or outpatient department≥1 |
|  | Or fasting glucose level ≥ 126 mg/dL | Index health examination |
| **Dyslipidemia** | E78 | Admission or outpatient department≥1 |
|  | Or Total cholesterol ≥ 240 mg/dL | Index health examination |
| **Heart failure** | I50 | Admission or outpatient department≥1 |
| **Prior stroke/TIA** | I63, I64 | Admission or outpatient department≥1 |
| **Peripheral artery disease** | I70, I73 | Admission or outpatient department≥1 |
| **Chronic kidney disease** | Estimated glomerular filtration rate <60 ml/min/1.73m^2^ | Index health examination |
| **Chronic obstructive pulmonary disease** | J41-44 | Admission or outpatient department≥1 |
| **Sleep apnea** | G473 | Admission or outpatient department≥1 |
| **Thyroid disease** | E05, E03 | Admission or outpatient department≥1 |
| **Health exam questionnaire** |  |  |
| **Smoking**  **Ex**  **Current** | Ex-smoker at the 1st examination and sustaining non-smoking till the 2nd examination  Current smoker at the 2nd examination regardless of the smoking status at the 1st examination. | Index health examination |
| **Alcohol consumption**  **Mild to moderate**  **Heavy** | Alcohol consumption >0g to <30g per day  Alcohol consumption ≥30g per day | Index health examination |
| **Regular exercise** | Performing a moderate physical activity more than 30 minutes at least 5 times per week or strenuous physical activity more than 20 minutes at least 3 times per week. | Index health examination |
| **Low income** | Income lowest 25% among the entire Korean population and supported by the medical aid | Index health examination |
| **Abdominal obesity** | Waist circumference of men≥90 cm or women≥85 cm | Index health examination |

Abbreviations: ICD, international classification of disease; CM, clinical modification; TIA, transient ischemic attack.

**Table S2. Baseline characteristics of the study population of combination therapy**

|  | **Combination** | | | | | | | | ***p-*value** |
| --- | --- | --- | --- | --- | --- | --- | --- | --- | --- |
|  | **ARB/BB**  **(n=1,595)** | | **ARB/CCB**  **(n=11,327)** | | **ARB/D**  **(n=14,632)** | | **ARB/CCB/D**  **(n=7,036)** | |  |
| **Age, years** |  |  | |  | |  | |  |  |
| **Mean ± SD** | 60.8 ± 11.0 | | 58.2 ± 11.5 | | 59. 5 ± 11.2 | | 60.3 ± 11.3 | | <0.001 |
| **< 65** | 982 (61.6) | | 8020 (70.8) | | 9826 (67.2) | | 4451 (63.3) | |  |
| **65–74** | 457 (28.7) | | 2387 (21.1) | | 3499 (23.9) | | 1859 (26.4) | |  |
| **≥ 75** | 156 (9.8) | | 920 (8.1) | | 1307 (8.9) | | 726 (10.3) | |  |
| **Sex (men)** | 861 (54.0) | | 6599 (58.3) | | 6906 (47.2) | | 3933 (55.9) | | <0.001 |
| **Comorbidities** |  |  | |  | |  | |  |  |
| **Diabetes mellitus** | 463 (29.0) | | 2971 (26.2) | | 3882 (26.5) | | 2219 (31.5) | | <0.001 |
| **Dyslipidaemia** | 947 (59.4) | | 4643 (41.0) | | 5943 (40.6) | | 2993 (42.5) | | <0.001 |
| **Heart failure** | 176 (11.0) | | 353 (3.1) | | 637 (4.4) | | 339 (4.8) | | <0.001 |
| **Prior ischemic stroke/TIA** | 109 (6.8) | | 730 (6.4) | | 730 (5.0) | | 473 (6.7) | | <0.001 |
| **Prior MI** | 149 (9.3) | | 71 (0.6) | | 94 (0.6) | | 50 (0.7) | | <0.001 |
| **PAD** | 333 (20.9) | | 1956 (17.3) | | 2728 (18.6) | | 1508 (21.4) | | <0.001 |
| **CKD** | 253 (15.9) | | 1224 (10.8) | | 2033 (13.9) | | 1108 (15.8) | | <0.001 |
| **COPD** | 156 (9.8) | | 965 (8.5) | | 1391 (9.5) | | 611 (8.7) | | 0.009 |
| **Sleep apnea** | 3 (0.2) | | 15 (0.1) | | 16 (0.1) | | 15 (0.2) | | 0.417 |
| **Thyroid disease** | 133 (8.3) | | 594 (5.2) | | 864 (5.9) | | 375 (5.3) | | <0.001 |
| **HTN duration, years** | 5.5 ± 3.1 | | 5.0 ± 3.5 | | 4.7 ± 3.2 | | 6.0 ± 3.0 | | <0.001 |
| **≥ 2yrs** | 1289 (80.8) | | 8279 (73.1) | | 10762 (73.6) | | 6089 (86.5) | | <0.001 |
| **Social history** |  |  | |  | |  | |  |  |
| **Smoking** |  |  | |  | |  | |  | <0.001 |
| **Non-smoker** | 993 (62.3) | | 6533 (57.7) | | 9569 (65.4) | | 4252 (60.4) | |  |
| **Ex-smoker** | 316 (19.8) | | 2263 (20.0) | | 2461 (16.8) | | 1370 (19.5) | |  |
| **Current smoker** | 286 (17.9) | | 2531 (22.3) | | 2602 (17.8) | | 1414 (20.1) | |  |
| **Alcohol consumption** |  |  | |  | |  | |  | <0.001 |
| **Non-drinker** | 1097 (68.8) | | 6198 (54.7) | | 9081 (62.1) | | 3907 (55.5) | |  |
| **Mild to moderate (0–30 g/day)** | 407 (25.5) | | 3885 (34.3) | | 4338 (29.7) | | 2379 (33.8) | |  |
| **Heavy (≥30g per day)** | 91 (5.7) | | 1244 (11.0) | | 1213 (8.3) | | 750 (10.7) | |  |
| **Regular exercise** | 294 (18.4) | | 2281 (20.1) | | 2920 (20.0) | | 1460 (20.8) | | 0.071 |
| **Low income** | 239 (15.0) | | 1758 (15.5) | | 2397 (16.4) | | 1171 (16.6) | | <0.001 |
| **Health examination** |  |  | |  | |  | |  |  |
| **SBP (mmHg)** | 131.2 ± 17.0 | | 134.5 ± 17 | | 132.9 ± 16.7 | | 133.9 ± 16.5 | | <0.001 |
| **DBP (mmHg)** | 80.0 ± 11.2 | | 82.4 ± 11.3 | | 81.8 ± 11.1 | | 81.6 ± 10.8 | | <0.001 |
| **BMI (kg/m^2^)** | 25.2 ± 3.2 | | 25.5 ± 3.3 | | 25.3 ± 3.3 | | 26.0 ± 3.4 | | <0.001 |
| **Obesity (BMI ≥25)** | 817 (51.2) | | 6112 (54.0) | | 7749 (53.0) | | 4221 (60.0) | | <0.001 |
| **WC (cm)** | 85.2 ± 8.6 | | 85.8 ± 8.7 | | 85.1 ± 8.6 | | 87.1 ± 8.6 | | <0.001 |
| **Abdominal obesity (Men≥90, Women≥85)** | 618 (38.8) | | 4563 (40.3) | | 5749 (39.3) | | 3308 (47.0) | | <0.001 |
| **Laboratory results** |  |  | |  | |  | |  |  |
| **eGFR (mL/min/1.73 m2)** | 80.1 ± 26.5 | | 84.8 ± 40.2 | | 82.5 ± 37.5 | | 81.3 ± 37.8 | | <0.001 |
| **Fasting Glucose (mg/dL)** | 108.5 ± 32.0 | | 108.5 ± 31.9 | | 108.6 ± 33.1 | | 110.8 ± 31.6 | | <0.001 |
| **Total cholesterol (mg/dL)** | 182.4 ± 41.5 | | 194.9 ± 39.3 | | 198.0 ± 38.5 | | 196.1 ± 38.4 | | <0.001 |
| **HDL-C (mg/dL)** | 50.6 ± 25.5 | | 52.8 ± 19.2 | | 52.9 ± 21.7 | | 52.9 ± 21.8 | | <0.001 |
| **LDL-C (mg/dL)** | 101.7 ± 36.9 | | 112.2 ± 39.5 | | 114.7 ± 38.9 | | 111.7 ± 37.9 | | <0.001 |
| ***TG (mg/dL)** | 136.5 (133.1– 140.0) | | 132.9 (131.6–134.2) | | 134.7 (133.5–135.9) | | 139.2 (137.5– 141.0) | | <0.001 |

Categorical variables were presented as a percentage and continuous variables were presented as mean and standard deviation.
*TG was presented as geometric mean (95% confidence interval).
Abbreviation: ACEi, Angiotensin-converting enzyme inhibitor; ARB, Angiotensin receptor blocker; BB, beta-blocker; BMI, body mass index; CCB, calcium channel blocker; CKD, chronic kidney disease; COPD, chronic obstructive pulmonary disease; D, diuretics; DBP, diastolic blood pressure; eGFR, estimated glomerular filtration rate; HDL-C, high density lipoprotein-cholesterol; LDL-C, low density lipoprotein-cholesterol; MI, myocardial infarction; PAD, peripheral artery disease; SBP, systolic blood pressure; TG, triglyceride; TIA, transient ischemic attack; WC, waist circumference.

**Table S3. Baseline characteristics of the study population according to specific ARB type in ARB monotherapy group**

|  | **Total**  **(n=14,778)** | **ARB type** | | | | | | | | ***p-*value** |
| --- | --- | --- | --- | --- | --- | --- | --- | --- | --- | --- |
|  |  | **Losartan (n=6,062)** | **Telmisartan (n=1,833)** | **Eprosartan (n=313)** | **Fimasartan (n=486)** | **Candesartan (n=1,420)** | **Irbesartan**  **(n=1,120)** | **Olmesartan**  **(n=2,043)** | **Valsartan**  **(n=1,501)** |  |
| **Age, years** |  |  |  |  |  |  |  |  |  | <0.001 |
| **Mean ± SD** | 58.0 ± 11.4 | 58.7 ± 11.4 | 57.1 ± 11.0 | 60.6 ± 10.8 | 55.2 ± 11.3 | 57.6 ± 11.5 | 58.3 ± 11.6 | 57.6 ± 11.2 | 57.4 ± 11.5 | <0.001 |
| **< 65** | 10539 (71.3) | 4182 (69.0) | 1367 (74.6) | 192 (61.3) | 387 (79.6) | 1031 (72.6) | 788 (70.4) | 1505 (73.7) | 1087 (72.4) | <0.001 |
| **65–74** | 3156 (21.4) | 1391 (23.0) | 368 (20.1) | 96 (30.7) | 70 (14.4) | 283 (19.9) | 249 (22.2) | 392 (19.2) | 307 (20.5) |  |
| **≥ 75** | 1083 (7.3) | 489 (8.1) | 98 (5.4) | 25 (8.0) | 29 (6.0) | 106 (7.5) | 83 (7.4) | 146 (7.2) | 107 (7.1) |  |
| **Sex (men)** | 7462 (50.5) | 2906 (47.9) | 953 (52.0) | 174 (55.6) | 260 (53.5) | 729 (51.3) | 578 (51.6) | 1050 (51.4) | 812 (54.1) | <0.001 |
| **Comorbidities** |  |  |  |  |  |  |  |  |  |  |
| **Diabetes mellitus** | 4380 (29.6) | 1746 (28.8) | 606 (33.1) | 97 (31.0) | 129 (26.5) | 448 (31.6) | 337 (30.1) | 563 (27.6) | 454 (30.3) | 0.002 |
| **Dyslipidaemia** | 6178 (41.8) | 2349 (38.8) | 840 (45.8) | 151 (48.2) | 205 (42.2) | 625 (44.0) | 497 (44.4) | 825 (40.4) | 686 (45.7) | <0.001 |
| **Heart failure** | 426 (2.9) | 184 (3.0) | 38 (2.1) | 12 (3.8) | 5 (1.0) | 52 (3.7) | 31 (2.8) | 41 (2.0) | 63 (4.2) | <0.001 |
| **Prior ischemic stroke/TIA** | 880 (6.0) | 262 (4.3) | 110 (6.0) | 60 (19.2) | 28 (5.8) | 98 (6.9) | 109 (9.7) | 128 (6.3) | 85 (5.7) | <0.001 |
| **Prior MI** | 131 (0.9) | 46 (0.8) | 15 (0.8) | 5 (1.6) | 0 (0.0) | 15 (1.1) | 11 (1.0) | 16 (0.8) | 23 (1.5) | 0.032 |
| **PAD** | 2281 (15.4) | 1049 (17.3) | 260 (14.2) | 50 (16.0) | 62 (12.8) | 199 (14.0) | 147 (13.1) | 312 (15.3) | 202 (13.5) | <0.001 |
| **CKD** | 1672 (11.3) | 672 (11.1) | 193 (10.5) | 43 (13.7) | 36 (7.4) | 146 (10.3) | 163 (14.6) | 256 (12.5) | 163 (10.9) | <0.001 |
| **COPD** | 1256 (8.5) | 520 (8.6) | 140 (7.6) | 36 (11.5) | 39 (8.0) | 126 (8.9) | 95 (8.5) | 159 (7.8) | 141 (9.4) | 0.273 |
| **Sleep apnea** | 22 (0.2) | 4 (0.1) | 7 (0.4) | 0 (0) | 1 (0.2) | 2 (0.1) | 2 (0.2) | 4 (0.2) | 2 (0.1) | 0.163 |
| **Thyroid disease** | 964 (6.5) | 357 (5.9) | 120 (6.6) | 22 (7.0) | 24 (4.9) | 108 (7.6) | 93 (8.3) | 140 (6.9) | 100 (6.7) | 0.034 |
| **HTN duration, years** | 3.9 ± 3.3 | 3.7 ± 3.3 | 4.1 ± 3.3 | 4.3 ± 3.2 | 3.3 ± 3.7 | 4.2 ± 3.4 | 4.8 ± 3.2 | 4.1 ± 3.2 | 4.0 ± 3.4 | <0.001 |
| **≥ 2yrs** | 9275 (62.8) | 3554 (58.6) | 1217 (66.4) | 215 (68.7) | 223 (45.9) | 925 (65.1) | 818 (73.0) | 1383 (67.7) | 940 (62.6) | <0.001 |
| **Social history** |  |  |  |  |  |  |  |  |  |  |
| **Smoking** | 9406 (63.7) | 3999 (66.0) | 1148 (62.6) | 200 (63.9) | 274 (56.4) | 894 (63.0) | 732 (65.4) | 1267 (62.0) | 892 (59.4) |  |
| **Non-smoker** | 2681 (18.1) | 1011 (16.7) | 362 (19.8) | 56 (17.89) | 65 (13.4) | 284 (20.0) | 200 (17.9) | 397 (19.4) | 306 (20.4) |  |
| **Ex-smoker** | 2691 (18.2) | 1052 (17.4) | 323 (17.6) | 57 (18.21) | 147 (30.3) | 242 (17.0) | 188 (16.8) | 379 (18.6) | 303 (20.2) |  |
| **Current smoker** |  |  |  |  |  |  |  |  |  | <0.001 |
| **Alcohol consumption** | 9122 (61.7) | 3859 (63.7) | 1082 (59.0) | 204 (65.18) | 249 (51.2) | 904 (63.7) | 696 (62.1) | 1220 (59.7) | 908 (60.5) |  |
| **Non-drinker** | 4528 (30.6) | 1759 (29.0) | 605 (33.0) | 91 (29.07) | 169 (34.8) | 418 (29.4) | 360 (32.1) | 662 (32.4) | 464 (30.9) |  |
| **Mild to moderate (0–30 g/day)** | 1128 (7.6) | 444 (7.3) | 146 (8.0) | 18 (5.75) | 68 (14.0) | 98 (6.9) | 64 (5.7) | 161 (7.9) | 129 (8.6) |  |
| **Heavy (≥30g per day)** | 3086 (20.9) | 1245 (20.5) | 392 (21.4) | 75 (23.96) | 93 (19.1) | 284 (20.0) | 244 (21.8) | 451 (22.1) | 302 (20.1) | 0.437 |
| **Regular exercise** | 9406 (63.7) | 3999 (66.0) | 1148 (62.6) | 200 (63.9) | 274 (56.4) | 894 (63.0) | 732 (65.4) | 1267 (62.0) | 892 (59.4) |  |
| **Low income** | 2268 (15.4) | 960 (15.8) | 257 (14.0) | 45 (14.4) | 97 (20.0) | 236 (16.6) | 163 (14.6) | 307 (15.0) | 203 (13.5) | 0.011 |
| **Health examination** |  |  |  |  |  |  |  |  |  |  |
| **SBP (mmHg)** | 132.2 ± 16.0 | 133.2 ± 15.8 | 131.9 ± 16.2 | 132.2 ± 15.9 | 134.6 ± 15.9 | 130.8 ± 16.5 | 130.8 ± 15.6 | 131.2 ± 16.5 | 131.7 ± 15.7 | <0.001 |
| **DBP (mmHg)** | 81.5 ± 10.9 | 82.0 ± 10.6 | 81.4 ± 10.6 | 80.5 ± 10.6 | 83.8 ± 11.5 | 80.4 ± 11.3 | 80.1 ± 10.6 | 81.2 ± 11.3 | 81.2 ± 10.9 | <0.001 |
| **BMI (kg/m^2^)** | 24.8 ± 3.2 | 24.6 ± 3.2 | 25.1 ± 3.2 | 24.6 ± 3.0 | 25.3 ± 3.6 | 24.9 ± 3.2 | 24.7 ± 3.1 | 24.9 ± 3.3 | 24.8 ± 3.1 | <0.001 |
| **Obesity (BMI ≥25)** | 6660 (45.1) | 2621 (43.2) | 908 (49.5) | 122 (39.0) | 244 (50.2) | 630 (44.4) | 502 (44.8) | 968 (47.4) | 665 (44.3) | <0.001 |
| **WC (cm)** | 83.8 ± 8.6 | 83.5 ± 8.6 | 84.3 ± 8.6 | 83.8 ± 8.0 | 85.1 ± 9.4 | 83.9 ± 8.6 | 83.8 ± 8.5 | 84.1 ± 8.6 | 83.9 ± 8.6 | <0.001 |
| **Abdominal obesity (Men≥90, Women≥85)** | 4823 (32.6) | 1934 (31.9) | 637 (34.8) | 91 (29.1) | 179 (36.8) | 463 (32.6) | 362 (32.3) | 698 (34.2) | 459 (30.6) | 0.024 |
| **Laboratory results** |  |  |  |  |  |  |  |  |  |  |
| **eGFR (mL/min/1.73 m2)** | 83.8 ± 30.1 | 83.8 ± 30.1 | 85.3 ± 47.6 | 82.1 ± 23.8 | 90.2 ± 54.2 | 83.9 ± 37.9 | 81.4 ± 26.0 | 83.2 ± 30.7 | 83.1 ± 23.3 | <0.001 |
| **Fasting Glucose (mg/dL)** | 109.3 ± 35.9 | 109.0 ± 36.9 | 110.6 ± 34.0 | 107.2 ± 35.7 | 112.0 ± 40.7 | 109.1 ± 33.8 | 109.1 ± 34.4 | 108.4 ± 33.5 | 110.2 ± 38.6 | 0.273 |
| **Total cholesterol (mg/dL)** | 194.8 ± 39.7 | 196.1 ± 39.0 | 195.2 ± 40.8 | 189.0 ± 39.7 | 197.5 ± 40.0 | 191.3 ± 40.4 | 188.3 ± 38.8 | 198.6 ± 40.0 | 192.7 ± 40.1 | <0.001 |
| **HDL-C (mg/dL)** | 53.5 ± 24.5 | 53.5 ± 20.7 | 53.2 ± 26.5 | 51.7 ± 12.9 | 54.2 ± 37.8 | 54.0 ± 34.3 | 51.7 ± 13.3 | 54.1 ± 27.2 | 54.0 ± 23.6 | 0.170 |
| **LDL-C (mg/dL)** | 112.9 ± 37.6 | 113.7 ± 35.8 | 112.8 ± 37.9 | 109.0 ± 37.0 | 113.5 ± 36.2 | 111.1± 38.8 | 109.0 ± 41.5 | 115.4 ± 40.2 | 111.3 ± 36.8 | <0.001 |
| ***TG (mg/dL)** | 127.4 (126.3–128.5) | 126.8 (125.1–128.6) | 130.1 (126.9–133.5) | 121.6 (114.4–129.2) | 140.7 (134.0–147.8) | 125.0 (121.6–128.6) | 122.4 (118.7–126.2) | 131.5 (128.4–134.7) | 123.9 (120.5–127.3) | <0.001 |

Categorical variables were presented as a percentage and continuous variables were presented as mean and standard deviation.
*TG was presented as geometric mean (95% confidence interval).
Abbreviation: BB, beta-blocker; BMI, body mass index; CCB, calcium channel blocker; CKD, chronic kidney disease; COPD, chronic obstructive pulmonary disease; D, diuretics; DBP, diastolic blood pressure; eGFR, estimated glomerular filtration rate; HDL-C, high density lipoprotein-cholesterol; LDL-C, low density lipoprotein-cholesterol; MI, myocardial infarction; PAD, peripheral artery disease; SBP, systolic blood pressure; TG, triglyceride; TIA, transient ischemic attack; WC, waist circumference.

**Table S4**. Hazard ratios for atrial fibrillation according to the antihypertensive medication type

| **Medication type** | **Number** | **Event** | **IR** | **Model 1**  **HR (95% CI)** | **Model 2**  **HR (95% CI)** | **Model 3**  **HR (95% CI)** | **Model 4**  **HR (95% CI)** |
| --- | --- | --- | --- | --- | --- | --- | --- |
| **Monotherapy** |  |  |  |  |  |  |  |
| ARB | 14778 | 475 | 4.26 | 1 (reference) | 1 (reference) | 1 (reference) | 1 (reference) |
| ACEi | 2072 | 102 | 6.23 | 1.44 (1.16–1.79) | 1.21 (0.98–1.50) | 1.20 (0.97–1.49) | 1.19 (0.96–1.47) |
| Beta-blocker | 13238 | 553 | 5.45 | 1.27 (1.13–1.44) | 1.48 (1.31–1.67) | 1.49 (1.31–1.69) | 1.51 (1.33–1.71) |
| CCB | 19882 | 775 | 5.00 | 1.16 (1.04–1.30) | 0.97 (0.87–1.09) | 1.00 (0.89–1.12) | 1.00 (0.89–1.12) |
| Diuretic | 8997 | 368 | 5.37 | 1.25 (1.09–1.43) | 1.31 (1.14–1.51) | 1.29 (1.13–1.49) | 1.37 (1.19–1.58) |
| ***p-*value** | | | | <0.001 | <0.001 | <0.001 | <0.001 |
| **Combination** |  |  |  |  |  |  |  |
| ARB/CCB | 11327 | 414 | 5.07 | 1 (reference) | 1 (reference) | 1 (reference) | 1 (reference) |
| ARB/BB | 1595 | 111 | 9.33 | 1.82 (1.47–2.24) | 1.63 (1.32–2.01) | 1.58 (1.28–1.96) | 1.54 (1.25–1.92) |
| ARB/D | 14632 | 570 | 5.09 | 0.99 (0.87–1.12) | 0.96 (0.85–1.09) | 0.96 (0.84–1.09) | 0.99 (0.87–1.12) |
| ARB/CCB/D | 7036 | 373 | 7.05 | 1.37 (1.19–1.58) | 1.25 (1.09–1.44) | 1.23 (1.07–1.41) | 1.18 (1.03–1.36) |
| ***p-*value** | | | | <0.001 | <0.001 | <0.001 | <0.001 |

IR is presented per 1000PY.

Multivariable adjusted model included age, sex, hypertension, diabetes mellitus, dyslipidemia, heart failure, prior ischemic stroke/transient ischemic attack, prior myocardial infarction, peripheral artery disease, chronic obstructive pulmonary disease, chronic kidney disease, sleep apnea, hyperthyroidism, and low income.

Model 1: unadjusted

Model 2: age and sex

Model 3: age, sex, diabetes mellitus, dyslipidemia, heart failure, prior ischemic stroke/transient ischemic attack, prior myocardial infarction, peripheral artery disease, chronic obstructive pulmonary disease, chronic kidney disease, sleep apnea, hyperthyroidism, drink, exercise, low income

Model 4: age, sex, diabetes mellitus, dyslipidemia, heart failure, prior ischemic stroke/transient ischemic attack, prior myocardial infarction, peripheral artery disease, chronic obstructive pulmonary disease, chronic kidney disease, sleep apnea, hyperthyroidism, drink, exercise, low income, systolic blood pressure, fasting glucose, total cholesterol, body mass index, hypertension duration

Abbreviations: ACEi, Angiotensin-converting enzyme inhibitor; ARB, Angiotensin receptor blocker; BB, beta-blocker; CI, confidence interval; D, diuretics; HR, hazard ratio; IR, incidence rate; PY, person-years.

**Table S5.** Hazard ratios for atrial fibrillation according to the specific ARB type

| **Medication type** | **Number** | **Event** | **IR** | **Model 1**  **HR (95% CI)** | **Model 2**  **HR (95% CI)** | **Model 3**  **HR (95% CI)** | **Model 4**  **HR (95% CI)** |
| --- | --- | --- | --- | --- | --- | --- | --- |
| **ARB only** | | | |  |  |  |  |
| Losartan | 6062 | 187 | 3.97 | 1 (reference) | 1 (reference) | 1 (reference) | 1 (reference) |
| Telmisartan | 1833 | 67 | 4.85 | 1.23 (0.93–1.69) | 1.36 (1.03–1.80) | 1.36 (1.03–1.80) | 1.35 (1.02–1.79) |
| Eprosartan | 313 | 12 | 4.84 | 1.21 (0.68–2.17) | 1.08 (0.60–1.94) | 1.00 (0.55–1.80) | 0.99 (0.55–1.79) |
| Fimasartan | 486 | 8 | 2.72 | 0.74 (0.37–1.51) | 0.91 (0.45–1.84) | 0.90 (0.44–1.84) | 0.91 (0.45–1.85) |
| Candesartan | 1420 | 58 | 5.62 | 1.45 (1.08–1.94) | 1.53 (1.14–2.06) | 1.50 (1.11–2.01) | 1.48 (1.10–1.99) |
| Irbesartan | 1120 | 43 | 5.07 | 1.29 (0.92–1.79) | 1.29 (0.93–1.80) | 1.25 (0.90–1.75) | 1.21 (0.87–1.70) |
| Olmesartan | 2043 | 52 | 3.34 | 0.85 (0.62–1.15) | 0.92 (0.67–1.25) | 0.90 (0.66–1.22) | 0.88 (0.65–1.21) |
| Valsartan | 1501 | 48 | 4.43 | 1.14 (0.83–1.57) | 1.21 (0.88–1.66) | 1.18 (0.86–1.62) | 1.16 (0.84–1.59) |
| ***p-*value** | | | | 0.074 | 0.048 | 0.063 | 0.082 |
| **ARB in combination with other antihypertension medications** | | | | | | | |
| Losartan | 22952 | 934 | 5.32 | 1 (reference) | 1 (reference) | 1 (reference) | 1 (reference) |
| Telmisartan | 7192 | 318 | 6.06 | 1.15 (1.02–1.31) | 1.27 (1.11–1.44) | 1.25 (1.10–1.42) | 1.20 (1.05–1.36) |
| Eprosartan | 1191 | 66 | 7.07 | 1.32 (1.03–1.69) | 1.23 (0.96–1.58) | 1.17 (0.91–1.51) | 1.15 (0.89–1.48) |
| Fimasartan | 625 | 13 | 3.49 | 0.71 (0.41–1.22) | 0.87 (0.50–1.51) | 0.85 (0.49–1.47) | 0.86 (0.50–1.49) |
| Candesartan | 4117 | 226 | 7.51 | 1.43 (1.23–1.65) | 1.44 (1.25–1.67) | 1.40 (1.21–1.62) | 1.33 (1.15–1.54) |
| Irbesartan | 3486 | 182 | 6.93 | 1.31 (1.11–1.53) | 1.29 (1.10–1.51) | 1.24 (1.06–1.46) | 1.16 (0.99–1.36) |
| Olmesartan | 7359 | 304 | 5.47 | 1.03 (0.91–1.17) | 1.10 (0.96–1.25) | 1.07 (0.94–1.22) | 1.05 (0.92–1.20) |
| Valsartan | 8139 | 349 | 5.96 | 1.14 (1.00–1.28) | 1.20 (1.06–1.36) | 1.17 (1.03–1.32) | 1.12 (0.99–1.26) |
| ***p-*value** | | | | <0.001 | <0.001 | <0.001 | 0.004 |

IR is presented per 1000PY.

Multivariable adjusted model included age, sex, hypertension, diabetes mellitus, dyslipidemia, heart failure, prior ischemic stroke/transient ischemic attack, prior myocardial infarction, peripheral artery disease, chronic obstructive pulmonary disease, chronic kidney disease, sleep apnea, hyperthyroidism, and low income.

Model 1: unadjusted

Model 2: age and sex

Model 3: age, sex, diabetes mellitus, dyslipidemia, heart failure, prior ischemic stroke/transient ischemic attack, prior myocardial infarction, peripheral artery disease, chronic obstructive pulmonary disease, chronic kidney disease, sleep apnea, hyperthyroidism, drink, exercise, low income

Model 4: age, sex, diabetes mellitus, dyslipidemia, heart failure, prior ischemic stroke/transient ischemic attack, prior myocardial infarction, peripheral artery disease, chronic obstructive pulmonary disease, chronic kidney disease, sleep apnea, hyperthyroidism, drink, exercise, low income, systolic blood pressure, fasting glucose, total cholesterol, body mass index, hypertension duration

Abbreviations: CI, confidence interval; HR, hazard ratio; IR, incidence rate; PY, person-years.

**Table S6**. Subgroup analyses according to antihypertensive medication type

| **Subgroup** | **Medication type** | **Number** | **Event** | **IR** | **Model 4**  **HR (95% CI)** | ***p* for interaction** |
| --- | --- | --- | --- | --- | --- | --- |
| **Monotherapy** |  |  |  |  |  |  |
| **Age** |  |  |  |  |  |  |
| **< 65** | **ARB** | 10539 | 216 | 2.66 | 1 (reference) | 0.034 |
|  | **ACEi** | 1292 | 32 | 2.99 | 0.94 (0.65–1.37) |  |
|  | **BB** | 9832 | 268 | 3.50 | 1.66 (1.39–1.99) |  |
|  | **CCB** | 11796 | 258 | 2.71 | 0.95 (0.79–1.13) |  |
|  | **Diuretic** | 6051 | 115 | 2.41 | 1.22 (0.97–1.54) |  |
| **65–74** | **ARB** | 3156 | 162 | 6.90 | 1 (reference) |  |
|  | **ACEi** | 584 | 54 | 12.17 | 1.60 (1.18–2.18) |  |
|  | **BB** | 2534 | 201 | 10.44 | 1.56 (1.27–1.93) |  |
|  | **CCB** | 5719 | 328 | 7.36 | 1.10 (0.91–1.33) |  |
|  | **Diuretic** | 2014 | 155 | 10.26 | 1.63 (1.30–2.04) |  |
| **≥ 75** | **ARB** | 1083 | 97 | 14.28 | 1 (reference) |  |
|  | **ACEi** | 196 | 16 | 12.99 | 0.88 (0.52–1.50) |  |
|  | **BB** | 872 | 84 | 14.91 | 1.12 (0.83–1.49) |  |
|  | **CCB** | 2367 | 189 | 12.41 | 0.92 (0.72–1.17) |  |
|  | **Diuretic** | 932 | 98 | 17.00 | 1.22 (0.92–1.62) |  |
| **Sex** |  |  |  |  |  |  |
| **Men** | **ARB** | 7462 | 248 | 4.43 | 1 (reference) | 0.159 |
|  | **ACEi** | 1184 | 59 | 6.36 | 1.17 (0.88–1.56) |  |
|  | **BB** | 5165 | 279 | 7.20 | 1.71 (1.44–2.04) |  |
|  | **CCB** | 8574 | 386 | 5.86 | 1.11 (0.94–1.30) |  |
|  | **Diuretic** | 2385 | 124 | 7.29 | 1.40 (1.13–1.75) |  |
| **Women** | **ARB** | 7316 | 227 | 4.08 | 1 (reference) |  |
|  | **ACEi** | 888 | 43 | 6.05 | 1.24 (0.90–1.72) |  |
|  | **BB** | 8073 | 274 | 4.37 | 1.32 (1.11–1.58) |  |
|  | **CCB** | 11308 | 389 | 4.36 | 0.90 (0.76–1.06) |  |
|  | **Diuretic** | 6612 | 244 | 4.74 | 1.30 (1.08–1.56) |  |
| **Body mass index** | | | | | | |
| **<25** | **ARB** | 8118 | 280 | 4.58 | 1 (reference) | 0.552 |
|  | **ACEi** | 1253 | 62 | 6.33 | 1.12 (0.85–1.47) |  |
|  | **BB** | 8561 | 325 | 4.98 | 1.40 (1.19–1.65) |  |
|  | **CCB** | 11495 | 462 | 5.23 | 0.98 (0.84–1.13) |  |
|  | **Diuretic** | 5497 | 208 | 5.04 | 1.27 (1.06–1.52) |  |
| **≥ 25** | **ARB** | 6660 | 195 | 3.87 | 1 (reference) |  |
|  | **ACEi** | 819 | 40 | 6.07 | 1.30 (0.92–1.82) |  |
|  | **BB** | 4677 | 228 | 6.30 | 1.68 (1.38–2.03) |  |
|  | **CCB** | 8387 | 313 | 4.69 | 1.03 (0.86–1.24) |  |
|  | **Diuretic** | 3500 | 160 | 5.87 | 1.52 (1.23–1.88) |  |
| **Abdominal obesity** | | | | | | |
| **No** | **ARB** | 9955 | 316 | 4.19 | 1 (reference) | 0.291 |
|  | **ACEi** | 1456 | 73 | 6.35 | 1.21 (0.94–1.57) |  |
|  | **BB** | 9930 | 358 | 4.69 | 1.38 (1.19–1.61) |  |
|  | **CCB** | 13538 | 495 | 4.69 | 0.95 (0.82–1.10) |  |
|  | **Diuretic** | 6373 | 225 | 4.63 | 1.27 (1.06–1.51) |  |
| **Yes** | **ARB** | 4823 | 159 | 4.40 | 1 (reference) |  |
|  | **ACEi** | 616 | 29 | 5.94 | 1.12 (0.75–1.66) |  |
|  | **BB** | 3308 | 195 | 7.73 | 1.77 (1.44–2.19) |  |
|  | **CCB** | 6344 | 280 | 5.64 | 1.10 (0.90–1.34) |  |
|  | **Diuretic** | 2624 | 143 | 7.18 | 1.56 (1.24–1.96) |  |
| **Smoking** |  |  |  |  |  |  |
| **Non** | **ARB** | 9406 | 302 | 4.21 | 1 (reference) | 0.869 |
|  | **ACEi** | 1254 | 69 | 6.93 | 1.34 (1.03–1.74) |  |
|  | **BB** | 9252 | 374 | 5.21 | 1.50 (1.29–1.75) |  |
|  | **CCB** | 13815 | 532 | 4.89 | 1.00 (0.86–1.15) |  |
|  | **Diuretic** | 7003 | 280 | 5.16 | 1.37 (1.16–1.62) |  |
| **Ex** | **ARB** | 2681 | 99 | 4.92 | 1 (reference) |  |
|  | **ACEi** | 438 | 17 | 4.95 | 0.82 (0.49–1.37) |  |
|  | **BB** | 1716 | 94 | 7.21 | 1.51 (1.14–2.00) |  |
|  | **CCB** | 2955 | 126 | 5.52 | 0.97 (0.74–1.26) |  |
|  | **Diuretic** | 775 | 40 | 7.11 | 1.24 (0.86–1.80) |  |
| **Current** | **ARB** | 2691 | 74 | 3.74 | 1 (reference) |  |
|  | **ACEi** | 380 | 16 | 5.36 | 1.16 (0.68–1.99) |  |
|  | **BB** | 2270 | 85 | 5.09 | 1.59 (1.16–2.17) |  |
|  | **CCB** | 3112 | 117 | 4.98 | 1.07 (0.80–1.43) |  |
| **Drinking** |  |  |  |  |  |  |
| **Non** | **ARB** | 9122 | 321 | 4.67 | 1 (reference) | 0.583 |
|  | **ACEi** | 1307 | 70 | 6.83 | 1.23 (0.95–1.60) |  |
|  | **BB** | 8902 | 388 | 5.67 | 1.44 (1.24–1.67) |  |
|  | **CCB** | 13154 | 516 | 5.04 | 0.94 (0.82–1.09) |  |
|  | **Diuretic** | 6496 | 294 | 5.93 | 1.38 (1.17–1.63) |  |
| **Mild to moderate** | **ARB** | 4528 | 124 | 3.59 | 1 (reference) |  |
|  | **ACEi** | 630 | 28 | 5.52 | 1.17 (0.77–1.76) |  |
|  | **BB** | 3689 | 133 | 4.70 | 1.63 (1.27–2.08) |  |
|  | **CCB** | 5368 | 198 | 4.68 | 1.10 (0.87–1.37) |  |
|  | **Diuretic** | 2078 | 58 | 3.65 | 1.24 (0.91–1.70) |  |
| **Heavy** | **ARB** | 1128 | 30 | 3.57 | 1 (reference) |  |
|  | **ACEi** | 135 | 4 | 3.78 | 0.78 (0.28–2.23) |  |
|  | **BB** | 647 | 32 | 6.63 | 1.94 (1.18–3.19) |  |
|  | **CCB** | 1360 | 61 | 5.81 | 1.26 (0.81–1.95) |  |
|  | **Diuretic** | 423 | 16 | 5.29 | 1.41 (0.77–2.58) |  |
|  | **Diuretic** | 1219 | 48 | 5.58 | 1.54 (1.07–2.22) |  |
| **Income** |  |  |  |  |  |  |
| **Others** | **ARB** | 12510 | 408 | 4.30 | 1 (reference) | 0.656 |
|  | **ACEi** | 1781 | 92 | 6.55 | 1.23 (0.98–1.54) |  |
|  | **BB** | 11118 | 465 | 5.44 | 1.48 (1.30–1.70) |  |
|  | **CCB** | 16768 | 657 | 5.01 | 0.98 (0.87–1.12) |  |
|  | **Diuretic** | 7377 | 310 | 5.50 | 1.38 (1.19–1.61) |  |
| **Low** | **ARB** | 2268 | 67 | 4.00 | 1 (reference) |  |
|  | **ACEi** | 291 | 10 | 4.28 | 0.90 (0.46–1.75) |  |
|  | **BB** | 2120 | 88 | 5.48 | 1.68 (1.22–2.31) |  |
|  | **CCB** | 3114 | 118 | 4.90 | 1.09 (0.81–1.48) |  |
|  | **Diuretic** | 1620 | 58 | 4.75 | 1.33 (0.94–1.90) |  |
| **SBP** |  |  |  |  |  |  |
| **< 130** | **ARB** | 5866 | 203 | 4.60 | 1 (reference) | 0.224 |
|  | **ACEi** | 905 | 49 | 6.90 | 1.30 (0.95–1.78) |  |
|  | **BB** | 7870 | 272 | 4.53 | 1.41 (1.17–1.70) |  |
|  | **CCB** | 7054 | 283 | 5.17 | 0.99 (0.82–1.19) |  |
|  | **Diuretic** | 5524 | 213 | 5.07 | 1.47 (1.21–1.79) |  |
| **≥ 130** | **ARB** | 8912 | 272 | 4.03 | 1 (reference) |  |
|  | **ACEi** | 1167 | 53 | 5.71 | 1.10 (0.82–1.48) |  |
|  | **BB** | 5368 | 281 | 6.78 | 1.62 (1.37–1.91) |  |
|  | **CCB** | 12828 | 492 | 4.90 | 1.01 (0.87–1.17) |  |
|  | **Diuretic** | 3473 | 155 | 5.85 | 1.25 (1.03–1.53) |  |
| **Hypertension duration** | | | | | | |
| **< 2years** | **ARB** | 5503 | 125 | 2.93 | 1 (reference) | 0.966 |
|  | **ACEi** | 519 | 16 | 3.79 | 1.00 (0.60–1.69) |  |
|  | **BB** | 7524 | 200 | 3.44 | 1.48 (1.18–1.85) |  |
|  | **CCB** | 5915 | 165 | 3.49 | 1.00 (0.79–1.27) |  |
|  | **Diuretic** | 5931 | 168 | 3.66 | 1.35 (1.07–1.71) |  |
| **≥ 2years** | **ARB** | 9275 | 350 | 5.08 | 1 (reference) |  |
|  | **ACEi** | 1553 | 86 | 7.07 | 1.23 (0.97–1.56) |  |
|  | **BB** | 5714 | 353 | 8.13 | 1.53 (1.31–1.77) |  |
|  | **CCB** | 13967 | 610 | 5.65 | 1.00 (0.88–1.14) |  |
|  | **Diuretic** | 3066 | 200 | 8.85 | 1.38 (1.15–1.64) |  |
| **Combination** |  |  |  |  |  |  |
| **Age** |  |  |  |  |  |  |
| **< 65** | **ARB/CCB** | 8020 | 187 | 3.17 | 1 (reference) | 0.398 |
|  | **ARB/BB** | 982 | 44 | 5.78 | 1.63 (1.17–2.27) |  |
|  | **ARB/D** | 9826 | 228 | 2.95 | 0.94 (0.78–1.15) |  |
|  | **ARB/CCB/D** | 4451 | 140 | 4.01 | 1.12 (0.90–1.39) |  |
| **65–74** | **ARB/CCB** | 2387 | 149 | 8.72 | 1 (reference) |  |
|  | **ARB/BB** | 457 | 53 | 15.88 | 1.73 (1.26–2.38) |  |
|  | **ARB/D** | 3499 | 242 | 9.16 | 1.09 (0.88–1.33) |  |
|  | **ARB/CCB/D** | 1859 | 151 | 11.05 | 1.19 (0.95–1.49) |  |
| **≥ 75** | **ARB/CCB** | 920 | 78 | 14.18 | 1 (reference) |  |
|  | **ARB/BB** | 156 | 14 | 14.73 | 0.98 (0.55–1.73) |  |
|  | **ARB/D** | 1307 | 100 | 12.21 | 0.89 (0.66–1.20) |  |
|  | **ARB/CCB/D** | 726 | 82 | 18.74 | 1.29 (0.94–1.76) |  |
| **Sex** |  |  |  |  |  |  |
| **Men** | **ARB/CCB** | 6599 | 235 | 4.95 | 1 (reference) | 0.695 |
|  | **ARB/BB** | 861 | 65 | 10.28 | 1.72 (1.30–2.28) |  |
|  | **ARB/D** | 6906 | 271 | 5.17 | 1.03 (0.86–1.23) |  |
|  | **ARB/CCB/D** | 3933 | 206 | 6.95 | 1.20 (1.00- 1.45) |  |
| **Women** | **ARB/CCB** | 4728 | 179 | 5.23 | 1 (reference) |  |
|  | **ARB/BB** | 734 | 46 | 8.25 | 1.34 (0.97–1.86) |  |
|  | **ARB/D** | 7726 | 299 | 5.02 | 0.94 (0.78–1.14) |  |
|  | **ARB/CCB/D** | 3103 | 167 | 7.16 | 1.15 (0.93–1.42) |  |
| **Obesity** | | | | | | |
| **BMI<25** | **ARB/CCB** | 5215 | 194 | 5.21 | 1 (reference) | 0.007 |
|  | **ARB/BB** | 778 | 55 | 9.53 | 1.63 (1.20–2.21) |  |
|  | **ARB/D** | 6883 | 291 | 5.59 | 1.10 (0.91–1.31) |  |
|  | **ARB/CCB/D** | 2815 | 124 | 5.94 | 0.99 (0.79–1.24) |  |
| **BMI ≥ 25** | **ARB/CCB** | 6112 | 220 | 4.95 | 1 (reference) |  |
|  | **ARB/BB** | 817 | 56 | 9.14 | 1.47 (1.09–1.98) |  |
|  | **ARB/D** | 7749 | 279 | 4.66 | 0.90 (0.75–1.07) |  |
|  | **ARB/CCB/D** | 4221 | 249 | 7.77 | 1.30 (1.09–1.56) |  |
| **Abdominal obesity** | | | | | | |
| **No** | **ARB/CCB** | 6764 | 222 | 4.53 | 1 (reference) | 0.014 |
|  | **ARB/BB** | 977 | 69 | 9.41 | 1.80 (1.36–2.36) |  |
|  | **ARB/D** | 8883 | 308 | 4.51 | 1.01 (0.85–1.20) |  |
|  | **ARB/CCB/D** | 3728 | 147 | 5.21 | 1.00 (0.81–1.24) |  |
| **Yes** | **ARB/CCB** | 4563 | 192 | 5.88 | 1 (reference) |  |
|  | **ARB/BB** | 618 | 42 | 9.20 | 1.26 (0.90–1.77) |  |
|  | **ARB/D** | 5749 | 262 | 5.99 | 0.97 (0.80–1.17) |  |
|  | **ARB/CCB/D** | 3308 | 226 | 9.14 | 1.34 (1.10–1.63) |  |
| **Smoking** |  |  |  |  |  |  |
| **Non** | **ARB/CCB** | 6533 | 252 | 5.30 | 1 (reference) | 0.487 |
|  | **ARB/BB** | 993 | 68 | 9.12 | 1.48 (1.13–1.94) |  |
|  | **ARB/D** | 9569 | 390 | 5.29 | 1.01 (0.86–1.18) |  |
|  | **ARB/CCB/D** | 4252 | 244 | 7.62 | 1.23 (1.03–1.46) |  |
| **Ex** | **ARB/CCB** | 2263 | 81 | 4.93 | 1 (reference) |  |
|  | **ARB/BB** | 316 | 29 | 12.39 | 2.19 (1.43–3.36) |  |
|  | **ARB/D** | 2461 | 92 | 4.93 | 1.00 (0.74–1.34) |  |
|  | **ARB/CCB/D** | 1370 | 76 | 7.28 | 1.25 (0.91–1.70) |  |
| **Current** | **ARB/CCB** | 2531 | 81 | 4.57 | 1 (reference) |  |
|  | **ARB/BB** | 286 | 14 | 6.66 | 1.12 (0.64–1.99) |  |
|  | **ARB/D** | 2602 | 88 | 4.49 | 0.93 (0.69–1.26) |  |
|  | **ARB/CCB/D** | 1414 | 53 | 5.05 | 0.96 (0.68–1.36) |  |
| **Drinking** |  |  |  |  |  |  |
| **Non** | **ARB/CCB** | 6198 | 253 | 5.69 | 1 (reference) | 0.409 |
|  | **ARB/BB** | 1097 | 77 | 9.39 | 1.46 (1.13–1.90) |  |
|  | **ARB/D** | 9081 | 387 | 5.59 | 0.98 (0.84–1.15) |  |
|  | **ARB/CCB/D** | 3907 | 238 | 8.20 | 1.24 (1.04–1.49) |  |
| **Mild to moderate** | **ARB/CCB** | 3885 | 115 | 4.05 | 1 (reference) |  |
|  | **ARB/BB** | 407 | 29 | 9.62 | 2.05 (1.36–3.09) |  |
|  | **ARB/D** | 4338 | 134 | 4.00 | 1.00 (0.78–1.28) |  |
|  | **ARB/CCB/D** | 2379 | 103 | 5.64 | 1.17 (0.90–1.53) |  |
| **Heavy** | **ARB/CCB** | 1244 | 46 | 5.24 | 1 (reference) |  |
|  | **ARB/BB** | 91 | 5 | 7.32 | 1.00 (0.40–2.52) |  |
|  | **ARB/D** | 1213 | 49 | 5.35 | 1.03 (0.69–1.54) |  |
|  | **ARB/CCB/D** | 750 | 32 | 5.64 | 0.88 (0.56–1.38) |  |
| **Income** |  |  |  |  |  |  |
| **Others** | **ARB/CCB** | 9569 | 351 | 5.06 | 1 (reference) | 0.813 |
|  | **ARB/BB** | 1356 | 94 | 9.27 | 1.53 (1.21–1.93) |  |
|  | **ARB/D** | 12235 | 478 | 5.09 | 0.99 (0.87–1.14) |  |
|  | **ARB/CCB/D** | 5865 | 317 | 7.17 | 1.21 (1.04–1.41) |  |
| **Low** | **ARB/CCB** | 1758 | 63 | 5.12 | 1 (reference) |  |
|  | **ARB/BB** | 239 | 17 | 9.65 | 1.64 (0.96–2.81) |  |
|  | **ARB/D** | 2397 | 92 | 5.12 | 0.96 (0.70–1.32) |  |
|  | **ARB/CCB/D** | 1171 | 56 | 6.42 | 1.03 (0.72–1.48) |  |
| **SBP** |  |  |  |  |  |  |
| **< 130** | **ARB/CCB** | 3988 | 165 | 5.75 | 1 (reference) | 0.012 |
|  | **ARB/BB** | 698 | 46 | 8.84 | 1.38 (0.99–1.93) |  |
|  | **ARB/D** | 5654 | 196 | 4.52 | 0.76 (0.62–0.93) |  |
|  | **ARB/CCB/D** | 2492 | 115 | 6.09 | 0.94 (0.74–1.19) |  |
| **≥ 130** | **ARB/CCB** | 7339 | 249 | 4.70 | 1 (reference) |  |
|  | **ARB/BB** | 897 | 65 | 9.71 | 1.64 (1.24–2.16) |  |
|  | **ARB/D** | 8978 | 374 | 5.46 | 1.15 (0.98–1.35) |  |
|  | **ARB/CCB/D** | 4544 | 258 | 7.57 | 1.34 (1.13–1.60) |  |
| **HTN duration** |  |  |  |  |  |  |
| **< 2yrs** | **ARB/CCB** | 3048 | 52 | 2.29 | 1 (reference) | 0.259 |
|  | **ARB/BB** | 306 | 16 | 6.73 | 2.47 (1.40–4.35) |  |
|  | **ARB/D** | 3870 | 98 | 3.21 | 1.26 (0.90–1.76) |  |
|  | **ARB/CCB/D** | 947 | 28 | 3.75 | 1.50 (0.95–2.37) |  |
| **≥ 2yrs** | **ARB/CCB** | 8279 | 362 | 6.14 | 1 (reference) |  |
|  | **ARB/BB** | 1289 | 95 | 9.98 | 1.44 (1.14–1.81) |  |
|  | **ARB/D** | 10762 | 472 | 5.80 | 0.95 (0.82–1.09) |  |
|  | **ARB/CCB/D** | 6089 | 345 | 7.59 | 1.14 (0.98–1.32) |  |

IR is presented per 1000PY.

Multivariable adjusted model included age, sex, hypertension, diabetes mellitus, dyslipidemia, heart failure, prior ischemic stroke/transient ischemic attack, prior myocardial infarction, peripheral artery disease, chronic obstructive pulmonary disease, chronic kidney disease, sleep apnea, hyperthyroidism, and low income.

Model 4: age, sex, diabetes mellitus, dyslipidemia, heart failure, prior ischemic stroke/transient ischemic attack, prior myocardial infarction, peripheral artery disease, chronic obstructive pulmonary disease, chronic kidney disease, sleep apnea, hyperthyroidism, drink, exercise, low income, systolic blood pressure, fasting glucose, total cholesterol, body mass index, hypertension duration.

Abbreviations: ACEi, Angiotensin-converting enzyme inhibitor; ARB, Angiotensin receptor blocker; BB, beta-blocker; BMI, body mass index; CI, confidence interval; D, diuretics; HR, hazard ratio; IR, incidence rate; PY, person-years.

**Table S7**. Subgroup analyses according to specific ARB type in ARB monotherapy

| **Subgroup** | **Medication type** | **Number** | **Event** | **IR** | **Model 4**  **HR (95% CI)** | ***p* for interaction** |  |  |  |  |
| --- | --- | --- | --- | --- | --- | --- | --- | --- | --- | --- |
| **Age** |  |  |  |  |  |  |  |  |  |  |
| **< 65** | Losartan | 4182 | 80 | 2.40 | 1 (reference) | 0.876 |  |  |  |  |
|  | Telmisartan | 1367 | 31 | 2.96 | 1.27 (0.84–1.93) |  |  |  |  |  |
|  | Eprosartan | 192 | 6 | 3.81 | 1.38 (0.60–3.17) |  |  |  |  |  |
|  | Fimasartan | 387 | 5 | 2.12 | 1.10 (0.45–2.73) |  |  |  |  |  |
|  | Candesartan | 1031 | 27 | 3.51 | 1.49 (0.96–2.31) |  |  |  |  |  |
|  | Irbesartan | 788 | 23 | 3.79 | 1.50 (0.94–2.40) |  |  |  |  |  |
|  | Olmesartan | 1505 | 26 | 2.21 | 0.91 (0.58–1.42) |  |  |  |  |  |
|  | Valsartan | 1087 | 18 | 2.25 | 0.95 (0.57–1.58) |  |  |  |  |  |
| **65–74** | Losartan | 1391 | 70 | 6.55 | 1 (reference) |  |  |  |  |  |
|  | Telmisartan | 368 | 26 | 9.58 | 1.45 (0.93–2.29) |  |  |  |  |  |
|  | Eprosartan | 96 | 4 | 5.47 | 0.75 (0.27–2.06) |  |  |  |  |  |
|  | Fimasartan | 70 | 3 | 7.10 | 1.26 (0.40–4.01) |  |  |  |  |  |
|  | Candesartan | 283 | 15 | 7.53 | 1.12 (0.64–1.97) |  |  |  |  |  |
|  | Irbesartan | 249 | 12 | 6.38 | 0.90 (0.49–1.67) |  |  |  |  |  |
|  | Olmesartan | 392 | 13 | 4.51 | 0.67 (0.37–1.22) |  |  |  |  |  |
|  | Valsartan | 307 | 19 | 8.74 | 1.34 (0.80–2.23) |  |  |  |  |  |
| **≥ 75** | Losartan | 489 | 37 | 11.88 | 1 (reference) |  |  |  |  |  |
|  | Telmisartan | 98 | 10 | 15.83 | 1.32 (0.66–2.66) |  |  |  |  |  |
|  | Eprosartan | 25 | 2 | 11.68 | 0.83 (0.20–3.47) |  |  |  |  |  |
|  | Fimasartan | 29 | 0 | 0.00 | - |  |  |  |  |  |
|  | Candesartan | 106 | 16 | 24.92 | 2.04 (1.13–3.71) |  |  |  |  |  |
|  | Irbesartan | 83 | 8 | 15.24 | 1.17 (0.54–2.51) |  |  |  |  |  |
|  | Olmesartan | 146 | 13 | 14.49 | 1.18 (0.63–2.22) |  |  |  |  |  |
|  | Valsartan | 107 | 11 | 16.85 | 1.33 (0.68–2.26) |  |  |  |  |  |
| **Sex** |  |  |  |  |  |  |  |  |  |  |
| **Men** | **Losartan** | 2906 | 96 | 4.27 | 1 (reference) | 0.333 |  |  |  |  |
|  | **Telmisartan** | 953 | 30 | 4.17 | 1.08 (0.71–1.623) |  |  |  |  |  |
|  | **Eprosartan** | 174 | 5 | 3.58 | 0.70 (0.28–1.736) |  |  |  |  |  |
|  | **Fimasartan** | 260 | 5 | 3.25 | 1.01 (0.41–2.488) |  |  |  |  |  |
|  | **Candesartan** | 729 | 36 | 6.86 | 1.71 (1.16–2.515) |  |  |  |  |  |
|  | **Irbesartan** | 578 | 25 | 5.84 | 1.27 (0.81–1.974) |  |  |  |  |  |
|  | **Olmesartan** | 1050 | 29 | 3.65 | 0.91 (0.60–1.376) |  |  |  |  |  |
|  | **Valsartan** | 812 | 22 | 3.76 | 0.96 (0.60–1.521) |  |  |  |  |  |
| **Women** | **Losartan** | 3156 | 91 | 3.69 | 1 (reference) |  |  |  |  |  |
|  | **Telmisartan** | 880 | 37 | 5.59 | 1.68 (1.15–2.47) |  |  |  |  |  |
|  | **Eprosartan** | 139 | 7 | 6.47 | 1.39 (0.65–3.01) |  |  |  |  |  |
|  | **Fimasartan** | 226 | 3 | 2.14 | 0.78 (0.25–2.46) |  |  |  |  |  |
|  | **Candesartan** | 691 | 22 | 4.34 | 1.20 (0.75–1.92) |  |  |  |  |  |
|  | **Irbesartan** | 542 | 18 | 4.28 | 1.14 (0.69–1.90) |  |  |  |  |  |
|  | **Olmesartan** | 993 | 23 | 3.02 | 0.86 (0.54–1.35) |  |  |  |  |  |
|  | **Valsartan** | 689 | 26 | 5.22 | 1.40 (0.90–2.17) |  |  |  |  |  |
| **Obesity** |  |  |  |  |  |  |  |  |  |  |
| **BMI < 25** | **Losartan** | 3441 | 113 | 4.22 | 1 (reference) | 0.905 |  |  |  |  |
|  | **Telmisartan** | 925 | 38 | 5.46 | 1.40 (0.97–2.03) |  |  |  |  |  |
|  | **Eprosartan** | 191 | 9 | 6.00 | 1.23 (0.62–2.44) |  |  |  |  |  |
|  | **Fimasartan** | 242 | 5 | 3.41 | 1.03 (0.42–2.54) |  |  |  |  |  |
|  | **Candesartan** | 790 | 33 | 5.83 | 1.47 (0.99–2.17) |  |  |  |  |  |
|  | **Irbesartan** | 618 | 26 | 5.58 | 1.31 (0.85–2.01) |  |  |  |  |  |
|  | **Olmesartan** | 1075 | 31 | 3.82 | 0.91 (0.61–1.36) |  |  |  |  |  |
|  | **Valsartan** | 836 | 25 | 4.16 | 1.01 (0.65–1.56) |  |  |  |  |  |
| **BMI ≥ 25** | **Losartan** | 2621 | 74 | 3.63 | 1 (reference) |  |  |  |  |  |
|  | **Telmisartan** | 908 | 29 | 4.23 | 1.28 (0.83–1.97) |  |  |  |  |  |
|  | **Eprosartan** | 122 | 3 | 3.07 | 0.63 (0.20–2.02) |  |  |  |  |  |
|  | **Fimasartan** | 244 | 3 | 2.04 | 0.76 (0.24–2.40) |  |  |  |  |  |
|  | **Candesartan** | 630 | 25 | 5.36 | 1.51 (0.96–2.38) |  |  |  |  |  |
|  | **Irbesartan** | 502 | 17 | 4.45 | 1.09 (0.64–1.86) |  |  |  |  |  |
|  | **Olmesartan** | 968 | 21 | 2.82 | 0.84 (0.52–1.37) |  |  |  |  |  |
|  | **Valsartan** | 665 | 23 | 4.76 | 1.37 (0.86–2.19) |  |  |  |  |  |
| **Abdominal obesity** | | | | | | |  |  |  | **Abdominal obesity** |
| **No** | **Losartan** | 4128 | 126 | 3.90 | 1 (reference) | 0.622 |  |  |  |  |
|  | **Telmisartan** | 1196 | 45 | 4.93 | 1.39 (0.99–1.96) |  |  |  |  |  |
|  | **Eprosartan** | 222 | 10 | 5.76 | 1.23 (0.65–2.36) |  |  |  |  |  |
|  | **Fimasartan** | 307 | 5 | 2.68 | 0.89 (0.36–2.18) |  |  |  |  |  |
|  | **Candesartan** | 957 | 36 | 5.18 | 1.42 (0.98–2.06) |  |  |  |  |  |
|  | **Irbesartan** | 758 | 31 | 5.38 | 1.36 (0.92–2.02) |  |  |  |  |  |
|  | **Olmesartan** | 1345 | 35 | 3.45 | 0.92 (0.63–1.34) |  |  |  |  |  |
|  | **Valsartan** | 1042 | 28 | 3.72 | 0.98 (0.65–1.48) |  |  |  |  |  |
| **Yes** | **Losartan** | 1934 | 61 | 4.10 | 1 (reference) |  |  |  |  |  |
|  | **Telmisartan** | 637 | 22 | 4.69 | 1.27 (0.78–2.07) |  |  |  |  |  |
|  | **Eprosartan** | 91 | 2 | 2.70 | 0.50 (0.12–2.07) |  |  |  |  |  |
|  | **Fimasartan** | 179 | 3 | 2.79 | 0.95 (0.30–3.03) |  |  |  |  |  |
|  | **Candesartan** | 463 | 22 | 6.53 | 1.58 (0.97–2.58) |  |  |  |  |  |
|  | **Irbesartan** | 362 | 12 | 4.42 | 0.95 (0.51–1.76) |  |  |  |  |  |
|  | **Olmesartan** | 698 | 17 | 3.15 | 0.81 (0.47–1.40) |  |  |  |  |  |
|  | **Valsartan** | 459 | 20 | 6.04 | 1.54 (0.93–2.56) |  |  |  |  |  |
| **Smoking** | | | | | | |  |  |  | **Smoking** |
| **Non** | **Losartan** | 3999 | 121 | 3.86 | 1 (reference) | 0.313 |  |  |  |  |
|  | **Telmisartan** | 1148 | 47 | 5.39 | 1.59 (1.13–2.22) |  |  |  |  |  |
|  | **Eprosartan** | 200 | 9 | 5.80 | 1.21 (0.61–2.39) |  |  |  |  |  |
|  | **Fimasartan** | 274 | 4 | 2.37 | 0.80 (0.30–2.17) |  |  |  |  |  |
|  | **Candesartan** | 894 | 33 | 5.05 | 1.33 (0.90–1.96) |  |  |  |  |  |
|  | **Irbesartan** | 732 | 24 | 4.25 | 1.06 (0.68–1.65) |  |  |  |  |  |
|  | **Olmesartan** | 1267 | 31 | 3.19 | 0.87 (0.58–1.29) |  |  |  |  |  |
|  | **Valsartan** | 892 | 33 | 5.09 | 1.35 (0.91–1.98) |  |  |  |  |  |
| **Ex** | **Losartan** | 1011 | 38 | 4.92 | 1 (reference) |  |  |  |  |  |
|  | **Telmisartan** | 362 | 15 | 5.64 | 1.21 (0.66–2.20) |  |  |  |  |  |
|  | **Eprosartan** | 56 | 2 | 4.38 | 0.76 (0.18–3.18) |  |  |  |  |  |
|  | **Fimasartan** | 65 | 0 | 0.00 | - |  |  |  |  |  |
|  | **Candesartan** | 284 | 17 | 8.31 | 1.85 (1.04–3.29) |  |  |  |  |  |
|  | **Irbesartan** | 200 | 7 | 4.64 | 0.88 (0.39–1.98) |  |  |  |  |  |
|  | **Olmesartan** | 397 | 11 | 3.56 | 0.78 (0.40–1.54) |  |  |  |  |  |
|  | **Valsartan** | 306 | 9 | 3.96 | 0.84 (0.41–1.74) |  |  |  |  |  |
| **Current** | **Losartan** | 1052 | 28 | 3.46 | 1 (reference) |  |  |  |  |  |
|  | **Telmisartan** | 323 | 5 | 2.05 | 0.64 (0.25–1.65) |  |  |  |  |  |
|  | **Eprosartan** | 57 | 1 | 2.14 | 0.48 (0.07–3.56) |  |  |  |  |  |
|  | **Fimasartan** | 147 | 4 | 4.53 | 1.68 (0.59–4.82) |  |  |  |  |  |
|  | **Candesartan** | 242 | 8 | 4.61 | 1.47 (0.67–3.23) |  |  |  |  |  |
|  | **Irbesartan** | 188 | 12 | 9.02 | 2.42 (1.23–4.79) |  |  |  |  |  |
|  | **Olmesartan** | 379 | 10 | 3.63 | 1.09 (0.53–2.24) |  |  |  |  |  |
|  | **Valsartan** | 303 | 6 | 2.88 | 0.92 (0.38–2.23) |  |  |  |  |  |
| **Drink** |  |  |  |  |  |  |  |  |  |  |
| **Non** | **Losartan** | 3859 | 124 | 4.15 | 1 (reference) | 0.796 |  |  |  |  |
|  | **Telmisartan** | 1082 | 41 | 5.04 | 1.32 (0.93–1.89) |  |  |  |  |  |
|  | **Eprosartan** | 204 | 8 | 5.01 | 1.03 (0.50–2.12) |  |  |  |  |  |
|  | **Fimasartan** | 249 | 6 | 4.03 | 1.24 (0.54–2.81) |  |  |  |  |  |
|  | **Candesartan** | 904 | 42 | 6.42 | 1.57 (1.10–2.23) |  |  |  |  |  |
|  | **Irbesartan** | 696 | 28 | 5.29 | 1.17 (0.77–1.77) |  |  |  |  |  |
|  | **Olmesartan** | 1220 | 37 | 4.01 | 0.99 (0.68–1.43) |  |  |  |  |  |
|  | **Valsartan** | 908 | 35 | 5.36 | 1.28 (0.88–1.87) |  |  |  |  |  |
| **Mild to moderate** | **Losartan** | 1759 | 51 | 3.69 | 1 (reference) |  |  |  |  |  |
|  | **Telmisartan** | 605 | 24 | 5.24 | 1.53 (0.94–2.48) |  |  |  |  |  |
|  | **Eprosartan** | 91 | 3 | 4.08 | 0.78 (0.24–2.49) |  |  |  |  |  |
|  | **Fimasartan** | 169 | 2 | 1.94 | 0.71 (0.17–2.91) |  |  |  |  |  |
|  | **Candesartan** | 418 | 12 | 3.92 | 1.13 (0.60–2.12) |  |  |  |  |  |
|  | **Irbesartan** | 360 | 11 | 4.05 | 1.09 (0.56–2.09) |  |  |  |  |  |
|  | **Olmesartan** | 662 | 10 | 1.94 | 0.56 (0.28–1.10) |  |  |  |  |  |
|  | **Valsartan** | 464 | 11 | 3.22 | 0.92 (0.48–1.78) |  |  |  |  |  |
| **Heavy** | **Losartan** | 444 | 12 | 3.45 | 1 (reference) |  |  |  |  |  |
|  | **Telmisartan** | 146 | 2 | 1.81 | 0.61 (0.14–2.72) |  |  |  |  |  |
|  | **Eprosartan** | 18 | 1 | 6.92 | 1.80 (0.23–13.94) |  |  |  |  |  |
|  | **Fimasartan** | 68 | 0 | 0.00 | - |  |  |  |  |  |
|  | **Candesartan** | 98 | 4 | 5.60 | 2.06 (0.66–6.39) |  |  |  |  |  |
|  | **Irbesartan** | 64 | 4 | 8.48 | 2.67 (0.86–8.32) |  |  |  |  |  |
|  | **Olmesartan** | 161 | 5 | 4.23 | 1.39 (0.49–3.96) |  |  |  |  |  |
|  | **Valsartan** | 129 | 2 | 2.25 | 0.83 (0.19–3.73) |  |  |  |  |  |
| **Income** |  |  |  |  |  |  |  |  |  |  |
| **Others** | **Losartan** | 5102 | 162 | 4.07 | 1 (reference) | 0.312 |  |  |  |  |
|  | **Telmisartan** | 1576 | 57 | 4.77 | 1.32 (0.97–1.79) |  |  |  |  |  |
|  | **Eprosartan** | 268 | 12 | 5.68 | 1.13 (0.63–2.04) |  |  |  |  |  |
|  | **Fimasartan** | 389 | 5 | 2.10 | 0.66 (0.27–1.61) |  |  |  |  |  |
|  | **Candesartan** | 1184 | 52 | 6.02 | 1.53 (1.12–2.10) |  |  |  |  |  |
|  | **Irbesartan** | 957 | 40 | 5.50 | 1.28 (0.90–1.82) |  |  |  |  |  |
|  | **Olmesartan** | 1736 | 43 | 3.24 | 0.85 (0.61–1.19) |  |  |  |  |  |
|  | **Valsartan** | 1298 | 37 | 3.93 | 1.02 (0.72–1.47) |  |  |  |  |  |
| **Low** | **Losartan** | 960 | 25 | 3.40 | 1 (reference) |  |  |  |  |  |
|  | **Telmisartan** | 257 | 10 | 5.36 | 1.54 (0.74–3.22) |  |  |  |  |  |
|  | **Eprosartan** | 45 | 0 | 0.00 | - |  |  |  |  |  |
|  | **Fimasartan** | 97 | 3 | 5.39 | 2.49 (0.75–8.27) |  |  |  |  |  |
|  | **Candesartan** | 236 | 6 | 3.56 | 1.13 (0.46–2.77) |  |  |  |  |  |
|  | **Irbesartan** | 163 | 3 | 2.48 | 0.71 (0.21–2.34) |  |  |  |  |  |
|  | **Olmesartan** | 307 | 9 | 3.91 | 1.10 (0.51–2.37) |  |  |  |  |  |
|  | **Valsartan** | 203 | 11 | 7.77 | 2.03 (1.00–4.13) |  |  |  |  |  |
| **Hypertension duration** | | | | | |  |  |  |  |  |
| **< 2years** | **Losartan** | 5102 | 162 | 2.51 | 1 (reference) | 0.488 |  |  |  |  |
|  | **Telmisartan** | 1576 | 57 | 3.59 | 1.32 (0.97–1.79) |  |  |  |  |  |
|  | **Eprosartan** | 268 | 12 | 2.40 | 1.13 (0.63–2.04) |  |  |  |  |  |
|  | **Fimasartan** | 389 | 5 | 2.99 | 0.66 (0.27–1.61) |  |  |  |  |  |
|  | **Candesartan** | 1184 | 52 | 3.51 | 1.53 (1.12–2.10) |  |  |  |  |  |
|  | **Irbesartan** | 957 | 40 | 4.36 | 1.28 (0.90–1.82) |  |  |  |  |  |
|  | **Olmesartan** | 1736 | 43 | 2.98 | 0.85 (0.61–1.19) |  |  |  |  |  |
|  | **Valsartan** | 1298 | 37 | 2.96 | 1.02 (0.72–1.47) |  |  |  |  |  |
| **≥ 2years** | **Losartan** | 960 | 25 | 5.07 | 1 (reference) |  |  |  |  |  |
|  | **Telmisartan** | 257 | 10 | 5.51 | 1.54 (0.74–3.22) |  |  |  |  |  |
|  | **Eprosartan** | 45 | 0 | 6.09 | - |  |  |  |  |  |
|  | **Fimasartan** | 97 | 3 | 2.37 | 2.49 (0.75–8.27) |  |  |  |  |  |
|  | **Candesartan** | 236 | 6 | 6.80 | 1.13 (0.46–2.77) |  |  |  |  |  |
|  | **Irbesartan** | 163 | 3 | 5.33 | 0.71 (0.21–2.34) |  |  |  |  |  |
|  | **Olmesartan** | 307 | 9 | 3.52 | 1.10 (0.51–2.37) |  |  |  |  |  |
|  | **Valsartan** | 203 | 11 | 5.31 | 2.03 (1.00–4.13) |  |  |  |  |  |

IR is presented per 1000PY.

Multivariable adjusted model included age, sex, hypertension, diabetes mellitus, dyslipidemia, heart failure, prior ischemic stroke/transient ischemic attack, prior myocardial infarction, peripheral artery disease, chronic obstructive pulmonary disease, chronic kidney disease, sleep apnea, hyperthyroidism, and low income.

Model 4: age, sex, diabetes mellitus, dyslipidemia, heart failure, prior ischemic stroke/transient ischemic attack, prior myocardial infarction, peripheral artery disease, chronic obstructive pulmonary disease, chronic kidney disease, sleep apnea, hyperthyroidism, drink, exercise, low income, systolic blood pressure, fasting glucose, total cholesterol, body mass index, hypertension duration

Abbreviations: ACEi, Angiotensin-converting enzyme inhibitor; ARB, Angiotensin receptor blocker; BB, beta-blocker; BMI, body mass index; CI, confidence interval; D, diuretics; HR, hazard ratio; IR, incidence rate; PY, person-years.

**Table S8. Sensitivity analyses of antihypertension medications: analyses on subjects without significant comorbidities**

| **Medication type** | **Number** | **Event** | **IR** | **Model 4**  **HR (95% CI)** | ***p-*value** |
| --- | --- | --- | --- | --- | --- |
| **Monotherapy** |  |  |  |  |  |
| ARB | 3576 | 60 | 2.18 | 1 (reference) | <0.001 |
| ACEi | 331 | 18 | 6.64 | 2.40 (1.41–4.08) |  |
| BB | 4739 | 110 | 3.02 | 1.85 (1.34–2.55) |  |
| CCB | 5606 | 150 | 3.37 | 1.17 (0.86–1.58) |  |
| Diuretic | 3298 | 73 | 2.86 | 1.59 (1.12–2.27) |  |
| **Combination therapy** | |  |  |  |  |
| ARB/CCB | 2944 | 64 | 2.98 | 1 (reference) | 0.023 |
| ARB/BB | 174 | 11 | 8.15 | 2.75 (1.44–5.24) |  |
| ARB/Diuretic | 3480 | 92 | 3.38 | 1.17 (0.85–1.61) |  |
| ARB/CCB/Diuretic | 1369 | 40 | 3.77 | 1.13 (0.76–1.69) |  |

IR, per 1000 person-years.

Model 4: age, sex, diabetes mellitus, dyslipidemia, heart failure, prior ischemic stroke/transient ischemic attack, prior myocardial infarction, peripheral artery disease, chronic obstructive pulmonary disease, chronic kidney disease, sleep apnea, hyperthyroidism, drink, exercise, low income, systolic blood pressure, fasting glucose, total cholesterol, body mass index, hypertension duration

Abbreviations: ACEi, Angiotensin-converting enzyme inhibitor; ARB, Angiotensin receptor blocker; BB, beta blocker; CCB, calcium channel blocker; CI, confidence interval; HR, hazard ratio; IR, incidence rate; PY, person-year

**Table S9. Sensitivity analyses of specific ARB type: analyses on subjects without significant comorbidities (diabetes mellitus, dyslipidaemia, prior myocardial infarction, heart failure, prior ischemic stroke/transient ischemic attack, peripheral artery disease, chronic kidney disease, chronic obstructive pulmonary disease, and thyroid diseases.**

| **Medication type** | **Number** | **Event** | **IR per 1000 PY** | **Model 4**  **HR (95% CI)** | ***p*-value** |
| --- | --- | --- | --- | --- | --- |
| **ARB Monotherapy** | | | | |  |
| Losartan | 1551 | 31 | 2.51 | 1 (reference) | 0.206 |
| Telmisartan | 412 | 11 | 3.54 | 1.49 (0.74–2.99) |  |
| Eprosartan | 48 | 2 | 5.22 | 2.12 (0.50–8.93) |  |
| Fimasartan | 138 | 1 | 1.19 | 0.53 (0.07–3.94) |  |
| Candesartan | 314 | 6 | 2.56 | 1.12 (0.47–2.71) |  |
| Irbesartan | 233 | 2 | 1.09 | 0.45 (0.11–1.90) |  |
| Olmesartan | 527 | 5 | 1.21 | 0.52 (0.20–1.34) |  |
| Valsartan | 353 | 2 | 0.77 | 0.30 (0.07–1.24) |  |
| **ARB Combination therapy** | | | | |  |
| Losartan | 5374 | 136 | 3.23 | 1 (reference) | 0.596 |
| Telmisartan | 1674 | 43 | 3.48 | 1.15 (0.82–1.63) |  |
| Eprosartan | 181 | 5 | 3.34 | 1.06 (0.43–2.58) |  |
| Fimasartan | 172 | 1 | 0.95 | 0.44 (0.06–3.11) |  |
| Candesartan | 788 | 22 | 3.70 | 1.22 (0.77–1.91) |  |
| Irbesartan | 622 | 10 | 2.05 | 0.67 (0.35–1.27) |  |
| Olmesartan | 1705 | 42 | 3.18 | 1.05 (0.74–1.49) |  |
| Valsartan | 1775 | 49 | 3.80 | 1.25 (0.90–1.74) |  |

IR, per 1000 PY.

Model 4: age, sex, diabetes mellitus, dyslipidemia, heart failure, prior ischemic stroke/transient ischemic attack, prior myocardial infarction, peripheral artery disease, chronic obstructive pulmonary disease, chronic kidney disease, sleep apnea, hyperthyroidism, drink, exercise, low income, systolic blood pressure, fasting glucose, total cholesterol, body mass index, hypertension duration

Abbreviations: ACEi, Angiotensin-converting enzyme inhibitor; ARB, Angiotensin receptor blocker; BB, beta-blocker; CI, confidence interval; HR, hazard ratio; IR, incidence rate; PY, person-year
